# Extracellular Vesicle-Driven Crosstalk between Legume Plants and Rhizobia: The Peribacteroid Space of Symbiosomes as a Protein Trafficking Interface

**DOI:** 10.1021/acs.jproteome.4c00444

**Published:** 2024-12-12

**Authors:** Paula Ayala-García, Irene Herrero-Gómez, Irene Jiménez-Guerrero, Viktoria Otto, Natalia Moreno-de Castro, Mathias Müsken, Lothar Jänsch, Marco van Ham, José-María Vinardell, Francisco Javier López-Baena, Francisco Javier Ollero, Francisco Pérez-Montaño, José Manuel Borrero-de Acuña

**Affiliations:** † Department of Microbiology, Faculty of Biology, Universidad de Sevilla, Av. de la Reina Mercedes 6, 41012 Sevilla, Spain; ‡ Institute of Microbiology, Technische Universität Braunschweig, Spielmannstr. 7, 38106 Braunschweig, Germany; § Central Facility for Microscopy, Helmholtz Centre for Infection Research, Inhoffenstraße 7, 38124 Braunschweig, Germany; ∥ Cellular Proteome Research, Helmholtz Centre for Infection Research, Inhoffenstraße 7, 38124 Braunschweig, Germany

**Keywords:** extracellular vesicles, rhizobium, legume, soybean, bean, symbiosis, bacteroid, peribacteroid space

## Abstract

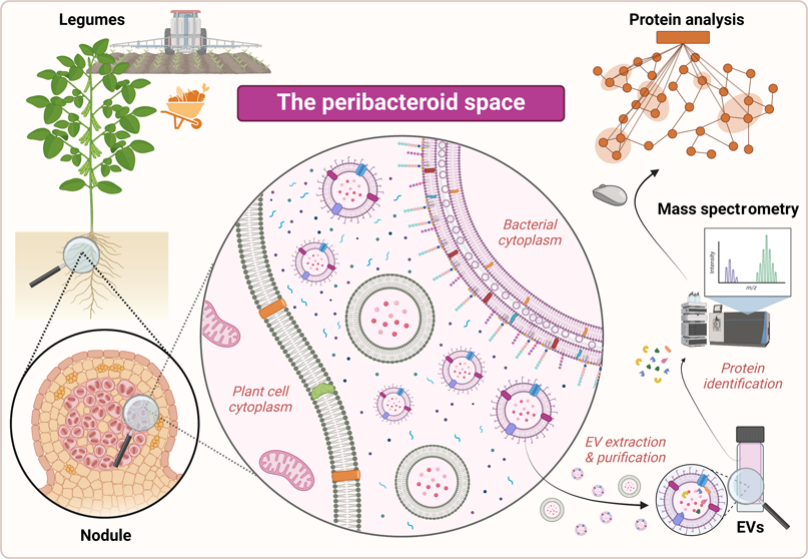

Prokaryotes and eukaryotes secrete extracellular vesicles (EVs) into the surrounding milieu to preserve and transport elevated concentrations of biomolecules across long distances. EVs encapsulate metabolites, DNA, RNA, and proteins, whose abundance and composition fluctuate depending on environmental cues. EVs are involved in eukaryote-to-prokaryote communication owing to their ability to navigate different ecological niches and exchange molecular cargo between the two domains. Among the different bacterium–host relationships, rhizobium–legume symbiosis is one of the closest known to nature. A crucial developmental stage of symbiosis is the formation of N_2_-fixing root nodules by the plant. These nodules contain endocytosed rhizobia—called bacteroids—confined by plant-derived peribacteroid membranes. The unrestricted interface between the bacterial external membrane and the peribacteroid membrane is the peribacteroid space. Many molecular aspects of symbiosis have been studied, but the interbacterial and interdomain molecule trafficking by EVs in the peribacteroid space has not been questioned yet. Here, we unveil intensive EV trafficking within the symbiosome interface of several rhizobium–legume dual systems by developing a robust EV isolation procedure. We analyze the EV-encased proteomes from the peribacteroid space of each bacterium–host partnership, uncovering both conserved and differential traits of every symbiotic system. This study opens the gates for designing EV-based biotechnological tools for sustainable agriculture.

## Introduction

Extracellular vesicles (EVs) are spherical mono- or bilayer lipidic structures of nanometer sizes (typically ranging from 20 to 400 nm) that are released into the external environment by organisms of all domains of life.^1^ In Gram-negative bacteria, EVs can originate either from explosive cell lysis or from blebs of the outer membrane. Regardless of the mechanism by which EVs are released into the extracellular space, they may originate from the outer membrane (OMV) or both the outer and inner membrane (OIMV).^1−4^ EVs contain a distinctive proteome within their cargoes in comparison to other cellular compartments. Thus, they are considered to represent an ancestral and unique bacterial secretion pathway: the type 0 secretion system (T0SS).^5^ In comparison to classic secretion systems that convey molecules into the extracellular space or directly translocate effectors or DNA into target cells, EVs seem to be additionally involved in the export of lipids, hydrophobic molecules, insoluble material, and virulence factors, among others.^6−8^ EV-based transport confers some benefits for the cargo, among which are synergistic activities of cocktail molecules, protection against degradation, and targeted delivery and maintenance of a critical concentration to ensure its activity.^3,9−12^ In fact, cargo compounds are concentrated in EVs, guaranteeing the delivery of a biologically relevant dose, or minimal critical concentration, into the target cells in a phenomenon referred to as quantal secretion.^1,3,8^ The delivery of the EV cargo into a target cell is ensured by membrane fusion or endocytosis. In contrast to quantal secretion, diffusion of any molecule, exported by secretion, transport systems, or any type of diffusion, entails a decrease in its concentration along the way to the target cell, thereby not ensuring that the concentration threshold of a given molecule to activate a biological process is maintained. EV-elicited biological processes affecting other organisms include bacterial killing, DNA transfer, effector delivery, immunomodulation, and bioactive compound trafficking.^13−17^ Most of these biological functions are especially relevant for pathogenic or symbiotic bacteria, including rhizobia.

Rhizobia are soilborne α- and ß-proteobacteria able to establish a symbiotic association with legumes to transform atmospheric nitrogen into assimilable ammonia inside specialized root organs called nodules, where rhizobia increase the nitrogen bioavailability to the benefit of the host plant, improving pulse performance.^18^ Rhizobia-orchestrated symbiosis fosters the growth and maturation of agronomically relevant legumes, avoiding the external input of nitrogen-rich fertilizers. Such agrochemical fertilizers are routinely used in extensive agriculture, which is a cornerstone to feed an ever-increasing population. Regrettably, these chemicals are environmentally hazardous as they contribute to aquifer eutrophication, soil degradation, and climate change with the associated economic losses.^19^ Shedding light on the EV-based symbiotic dialogue in agriculture-relevant plants can be harnessed to develop sustainable tools for crop growth promotion and/or plant immunization for upcoming infections.^20^ Among leguminous plants, soybean and bean, bear enormous agricultural importance worldwide, as they produced approximately 360 and 60 million metric tons of grain in 2022, respectively (source: http://www.fao.org/faostat/en/). The rhizobium–legume symbiotic interaction is established by a complex molecular dialogue whose onset is determined by the exudation of flavonoids by legume roots into the rhizosphere. These molecules are recognized by rhizobia, which activate the expression of the nodulation genes.^21,22^ Such genes encode proteins involved in the synthesis and export of compatible lipo-chitooligosaccharides molecules, also known as Nod factors (NFs).^23^ NFs are subsequently recognized by specific plant LysM receptor-like kinases, triggering a downstream transduction cascade that involves the upregulation of several symbiotic plant genes. Consequently, plants create an infection tube through which bacteria penetrate and travel, remaining outside of host cells (see the abstract Figure).^24^ Simultaneously, as infection threads progress, new plant organs, called nodules, develop in the root cortex. These developing nodules intersect with the dividing and branching infection threads.^25^ Eventually, rhizobia are endocytosed into the cytoplasm of nodule symbiotic cells, where they evolve into a nitrogen-fixing form known as a bacteroid (see the abstract Figure). Such bacteroids become constrained within membranes derived from plant cells, forming what is termed a symbiosome. Thus, the symbiosomes encompass the plant membrane and the bacteroid. The interface between both membranes is designated as the symbiosome or peribacteroid space.^25,26^ This shared peribacteroid space is the most intimate area in the rhizobium–legume symbiosis, a unique interkingdom molecular trafficking interface in nature. Moreover, since each symbiosome of determinate nodules (such as those formed by *Glycine max*, *Lotus burttii*, and *Phaseolus vulgaris*) contain several bacteroids,^27^ in this case, the peribacteroid space might also be an appropriate scenario for interbacterial molecular trafficking mediated by EVs. Several studies have highlighted the essential role of the EVs in bacterial–host interactions since these molecular conveyors can be internalized into eukaryotic cells to release their content, operating as communicating vessels.^2,5,9,28^ Although the basis for rhizobium–legume symbiosis has been investigated in depth over decades, the involvement of EVs during the infective process remains elusive. Bacterial EV-plant communication has recently been proposed to add an additional layer of complexity to the molecular dialogue of the plant holobiont.^29^ Along these lines, recent studies have demonstrated that the EVs isolated from free-living *Rhizobium etli* and *Sinorhizobium fredii* grown in the presence of nodulation gene-inducing flavonoids were able to deform the root hairs of host legumes, pointing out the presence of biologically active NFs inside these lipidic vehicles.^30,31^ However, the role of rhizobial EVs and the associated protein content in the late stages of the symbiotic process, when the host–bacterium relationship becomes most intimate, remains completely unexplored.

Here, we pipelined a protocol to isolate EVs from the peribacteroid space, containing rhizobial EVs and also exosomes, microvesicles, and apoptotic bodies of plants from different symbiotic pairs. Thus, the term peribacteroid EVs henceforth will refer to both vesicles from plant and bacterial origin. The procedure is suitable for the analysis and characterization of the EVs on different omics and biochemical levels. In this work, we specifically used this protocol to extensively analyze the proteome of these molecular containers isolated from unrestricted intimate environments, the peribacteroid space of two symbiotic partners of agricultural importance: *G. max*-*S. fredii* HH103 and *P. vulgaris*-*Rhizobium tropici* CIAT 899; and in the model legume *Lotus burttii*, that is nodulated by both HH103 and CIAT 899 strains. While the EV-proteome appears to be largely host-specific in the two rhizobial strains, only a few are conserved across partners; the biological processes, molecular functions, and cellular components affected were similar in all symbiotic pairs analyzed. Finally, we report a manifold of EV-associated proteins for each rhizobial strain with relevant functions for the development of the symbiotic process.

## Materials and Methods

### Bacterial Strains, Growth Conditions, and Nodule Harvesting

*S. fredii* HH103 (hereafter HH103) and *R. tropici* CIAT 899 (hereafter CIAT 899) strains were grown at 28 °C on modified yeast extract mannitol (YM3, with 3 g mL^–1^ of mannitol) medium.^32^ To obtain colonized nodules, the HH103 and CIAT 899 strains were grown in YM3 medium until the bacterial concentration reached about 10^9^ cells per mL^–1^. Surface-sterilized seeds of *G. max* (soybean), *P. vulgaris* (common bean), and *L. burttii* were pregerminated and placed on sterilized jars containing vermiculite and perlite substrates (3:1) and Fahraeus N-free solution as previously described.^32−35^ Each pregerminated and sterilized seed was inoculated with 1 mL of bacterial culture. Growth conditions were 16 h at 26 °C in the light and 8 h and 18 °C in the dark, with 70% of humidity. After 30 (soybean and common bean) or 50 (*L. burttii*) days postinoculation, around 100 root nodules from at least 3 different Leonard jars were harvested and weighed for each symbiotic partner.

### Isolation and Purification of EVs from the Peribacteroid Space

Isolation of EVs from peribacteroid space was performed following the protocol described by Ayala-García et al., with modifications.^36^ Nodules were ground with a sterilized pestle and mortar in MMS buffer [40 mM 3-(4-morpholino)-propanesulfonic acid, 20 mM KOH, 2 mM MgSO_4_, 0.3 M sucrose, pH 7.0], and the liquid was collected with a pipet. Debris derived from plant tissues was removed by passing them through a 40-μm cell strainer cap (BD Falcon, BD Biosciences, USA). Symbiosome-enriched eluents were centrifuged at 2200*g* for 5 min to remove rhizobial cells. The resulting supernatant fractions, containing proteins, metabolites, and EVs from the peribacteroid space, were centrifuged at 7000*g* and 4 °C for 20 min and filtered through a microporous membrane (0.22 μm) to remove any remaining rhizobium cells and cellular debris. To pellet EVs, filtered eluents were transferred to a sterile ultracentrifugation tube and ultracentrifuged, using a Himac P70AT-1759 Rotor (Ultracentrifuge CP 90NX, Eppendorf Himac Technologies, Japan), at 150 000*g* at 4 °C for 2 h. Pelleted EVs from the peribacteroid space were washed once with PBS buffer to remove the liquid medium and soluble proteins, resuspended in PBS buffer, and stored at 4 °C.

### Integrity and Quantification of EVs

Integrity and quantification assessment of purified EVs was performed following a previously described protocol.^37^ For transmission electron microscopy (TEM), a fine carbon film was positioned over 30–50 μL sample droplets to facilitate the adherence of vesicles. After 1 min, a 300-mesh copper grid was employed to lift the carbon film. The grid underwent two washes with droplets of distilled water before being exposed to a droplet containing 4% uranyl acetate (w/v). After 1 min, excess liquid was thoroughly removed using filter paper, and the grids were dried using a 60 W light bulb. Subsequently, the samples were scrutinized using a Libra 120 transmission electron microscope (Zeiss, Oberkochen, Germany) operating at an acceleration voltage of 120 kV and calibrated magnifications. Adjustments in contrast, brightness, and size measurements were carried out using WinTEM software v01.06.

EV yields were quantified by two approaches: lipid fluorescence and scattering-light-reliant nanoparticle tracking analysis.

EV yield quantification based on fluorescence was measured by monitoring the signal emitted by the membrane lipid dye FM1–43 (Invitrogen, Thermo Fisher Scientific, USA, *N*-(3-triethylammoniumpropyl)-4-(4-(dibutylamino)styryl) pyridinium dibromide). Stock solutions of FM1–43 were initially prepared in 20 mM HEPES buffer at a concentration of 50 μg mL^–1^ and stored at 4 °C. Prior to each measurement, a working solution for staining was freshly prepared and kept chilled on ice: the stock solution was diluted to a final concentration of 5 μg mL^–1^ in 20 mM HEPES buffer. Staining was conducted by using a 100-fold dilution of the sample in the prepared working solution. Subsequently, 200 μL of the stained solution was transferred in triplicate into individual wells of a 96-well flat-black-bottom plate (Thermo Fisher Scientific, USA). EVs were incubated for 30 min at 37 °C. As a blank, the PBS buffer was measured under the same conditions. Fluorescence measurements were promptly taken using a Synergy HT microtiter reader (Biotek, USA) under the following parameters: excitation at 500 nm, emission at 560 nm, gain set to 100 and at room temperature. Data were collected from 3 biological replicates.

Nanoparticle tracking analysis for EVs quantification was performed using the NanoSight NS300 nanoparticle analyzer (Malvern Panalytical, United Kingdom), applying a monochromatic laser beam at 488 nm and taking 90 s videos at 23 °C. To quantify and size EVs, each video was analyzed with the nanoparticle tracking analysis software following the manufacturer’s instructions. All particle counts were normalized to grams of nodule.

### Protein Profile Analysis and Proteomic Studies

Protein concentration was measured with a BCA kit according to the manufacturer’s instructions (Thermo Fisher Scientific, USA). Nonionic and nondenaturing surfactant Triton X-100 was added at 2% w/v final concentration to the samples to lyse the phospholipid bilayer(s) of EVs. EV aliquots were resuspended in sample buffer with a final concentration of 62.5 mM Tris-HCl (pH 6.8), 2% SDS (w/v), 10% glycerol (v/v), 5% ß-mercaptoethanol (w/v), and 0.001% bromophenol blue [w/v]. The same concentration (approximately 2 μg) of EV proteins was loaded in each lane, and proteins were separated by SDS-PAGE using the discontinuous buffer system of Laemmli.^38^ Electrophoresis was performed on SDS polyacrylamide gels (15%, w/v), and proteins were visualized by silver staining. Thereby, protein patterns derived from each EV sample were compared.

The protein digestion and peptide purification for LC-MS/MS followed a modified SP3 protocol.^39^ Initially, proteins underwent reduction using 5 mM TCEP and alkylation with 10 mM MMTS. These treated proteins were allowed to bind to SP3 carboxylate beads overnight in 60% acetonitrile (v/v). The beads were subsequently washed twice with 70% (v/v) ethanol and once with 100% (v/v) acetonitrile (v/v). Protein digestion occurred overnight at 37 °C using trypsin at a final concentration of 1 μg of protease per 50 μg of total protein in the presence of 50 mM TEAB, 5 mM TCEP, and 10 mM MMTS. After elution from the beads by using 2% DMSO (v/v), dried peptides were suspended in 50 μL of 0.1% formic acid (v/v) and allowed to resolve using an ultrasonic water bath. Samples were centrifuged for 20 min at 20 000*g* at 20 °C, and supernatants were transferred to new LoBind tubes. For each sample, 200 ng of peptide mixture was applied to C18 Evotips (EV-2001; Evosep, Denmark) according to the manufacturer’s protocol. Evotips were then loaded on an Evosep One HPLC instrument (Evosep) connected to a TimsTOF Pro mass spectrometer (Bruker, Spain). The Evosep One HPLC was operated with the standard 60 samples per day method (21 min gradient at a flow rate of 1.0 μL/min; buffer A: 0.1% formic acid (v/v); buffer B: 0.1% formic acid in acetonitrile (v/v)). For the TimsTOF*Pro* mass spectrometer, the standard MSMS Bruker method, “DDA PASEF method for short gradients with 0.5 s cycle time”, was employed. MS settings in detail were as follows: scan begin: 100 *m*/*z*; scan end: 1700 *m*/*z*; ion polarity: positive; scan mode: PASEF. Tims settings were: mode custom; number of PASEF ramps: 4; charge minimum: 0; charge maximum: 5; 1/K0 start: 0.75 V*s/cm^2^; 1/K0 end: 1.4 V*s/cm^2^; ramp time: 100.0 ms; MS average: 1. Raw data files were analyzed using PEAKS software (PEAKS studio version 10.6). Detailed PEAKS settings were as follows: fragmentation mode: high energy CID (y and b ions); parent mass error tolerance: 20.0 ppm; fragment mass error tolerance: 0.03 Da; enzyme: trypsin; max. missed cleavages: 1; fixed-modifications: β-methylthiolation C (45.99); variable modifications: oxidation M (15.99); false discovery rate (FDR): 1%; database: *S. fredii* HH103 and *Rhizobium tropici* CIAT 899. The experiments were conducted in three independent biological replicates. Proteins that were detected in at least two biological replicates for each condition were considered reliable. The mass spectrometry proteomics data have been deposited to the ProteomeXchange Consortium via the PRIDE [1] partner repository with the data set identifier PXD056185.

### Additional Bioinformatics Tools

BlastP analyses were performed on the NCBI server against the nonredundant protein sequences (nr) database to search for EV protein homologues. We selected the organisms *S. fredii* HH103 (NCBI txid1117943) and *R. tropici* CIAT 899 (NCBI txid698761) as default parameters. Gene ontology (GO) annotations were done using Blast2GO software v5.2 (https://www.blast2go.com/). Venn diagrams were carried out using the following server: https://bioinformatics.psb.ugent.be/webtools/Venn/.

The presence of signal peptides and the subcellular localization of proteins were predicted by submitting the protein FASTA sequences to the SignalP v6.0,^40^ SecretomeP v2.0,^41^ and PSORTb v3.0.2^42^ web server. Likewise, the transmembrane regions of proteins were predicted by the DeepTMHMM server.^43^

## Results and Discussion

### Peribacteroid Space from *G. max*, *P. vulgaris*, and *L. burttii* Nodules is Enriched with EVs

**Figure 1:**
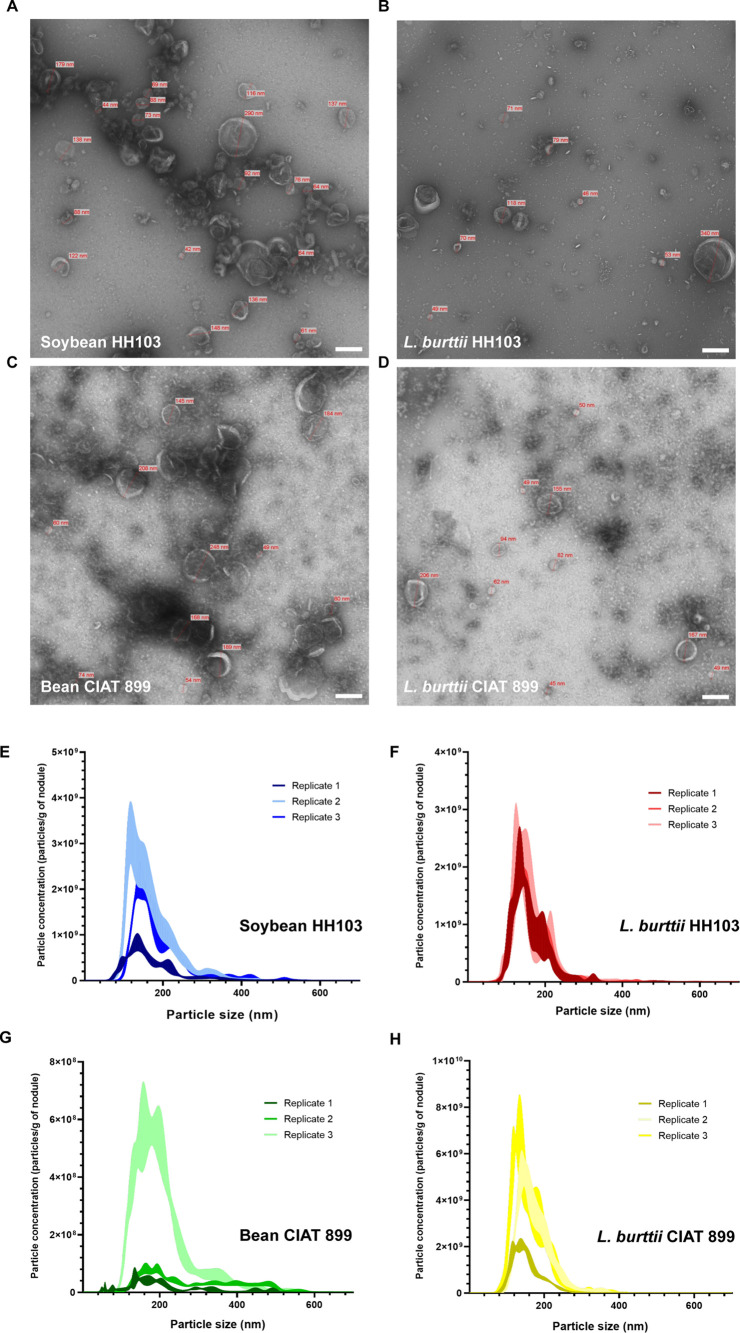

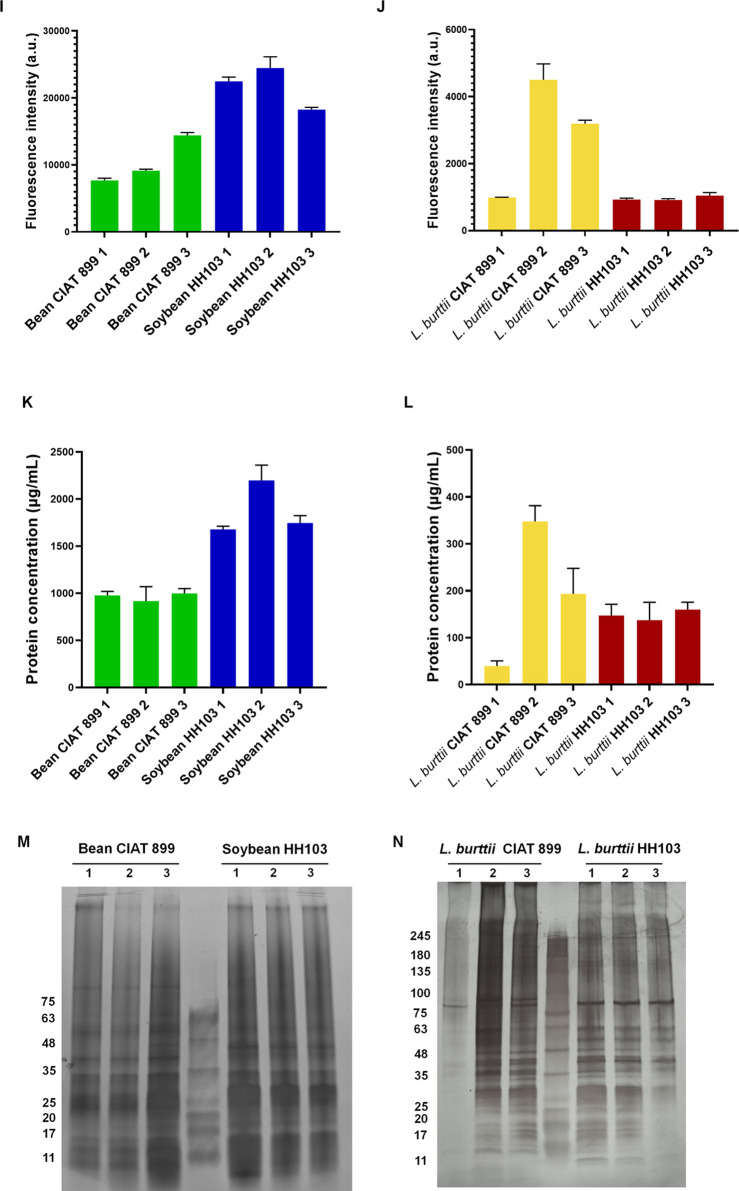
Analysis of peribacteroid EVs isolated from nodules induced by *S. fredii* HH103 and *R. tropici* CIAT 899 in different legume plants. (A–D) Transmission electron microscopy images of negatively stained EVs from the symbiotic pairs HH103-soybean (A), HH103-*L. burttii* (B), CIAT 899-bean (C), and CIAT 899-*L. burttii* (D). Scale bar = 200 nm. (E–H) Nanoparticle tracking analysis by the NanoSight system of EVs from the symbiotic pairs HH103-soybean (E), HH103-*L. burttii* (F), CIAT 899-bean (G), and CIAT 899-*L. burttii* (H). Three replicates were performed for each set of samples. Fluorescence intensity (in arbitrary units, au) of EVs stained with FM1–43 from the symbiotic pairs are represented in graphs (bean-CIAT 899 and soybean-HH103 pairs (I) and *L. burttii*-CIAT 899 and *L. burttii-*HH103 pairs (J)). Protein quantification was analyzed by the BCA assay of bacteroid EVs from the different symbiotic pairs CIAT 899-bean and HH103-soybean (K) and CIAT 899-*L. burttii* and HH103-*L. burttii* (L). Protein profiles of bacteroid EVs from the different symbiotic pairs were analyzed by silver staining (M, N). The molecular weight marker (kDa) is indicated on the left. The sizes of the scale bars are indicated in the electron microscopy figures. Vesicle diameters are represented.

To gain insights into the functions of EVs secreted by HH103 and CIAT 899 during the later stages of symbiosis, particularly when bacteria undergo a transformation into bacteroids, we initially isolated and purified EVs from the peribacteroid spaces of soybean (HH103), bean (CIAT 899), and *L. burttii* (both rhizobia) nodules. Transmission electron microscopy of EVs from bacteroids displayed distinct membranous nanostructures ranging from 20 to 400 nm in diameter in the purified samples from all four types of nodule symbiosomes. This indicated that the collected peribacteroid samples were devoid of bacterial cells and were enriched in EVs (Figure 1A–D). Considering the wide variety of vesicle sizes found in the samples, which frequently exceeded the bacterial known range (20–400 nm) but not that of the so-called eukaryotic microvesicles (50–2000 nm), it was reasonable to presume that these EVs could potentially originate from both partners involved in the symbiotic relationship.^17,44^ In fact, our proteomic analyses using the eukaryotic host databases revealed a significant number of plant-derived proteins; however, this is not in the scope of this study. For further consideration, we provide the EV-proteomic data set of soybean and bean Leguminosae plants in Supporting Tables 5 and 6, respectively. Unfortunately, the *L. burttii* genome is currently unavailable, and thus, the proteomic analysis was not conducted.

To assess the size distribution and total quantity of membranous particles present in the peribacteroid space in the three plants inoculated with HH103 and/or CIAT 899, we conducted nanoparticle tracking analysis using a light scattering system, the NanoSight analyzer. The obtained results revealed that the most frequently measured particle size ± standard error ranged from 134.5 ± 3.4 nm for EVs from the soybean-HH103 pair (Figure 1E) and 135.8 ± 4.9 nm for EVs from the *L. burttii-*HH103 pair (Figure 1F). Likewise, EVs from bean and *L. burttii* inoculated with CIAT 899 were measured around 167.3 ± 7.1 nm (Figure 1G) and 136.0 ± 6.3 nm (Figure 1H), respectively. These results are in agreement with the known size spectrum of EVs from Gram-negative bacteria and also with that of eukaryotic exosomes (30–150 nm).^1,4,44^ This protocol is thereby suited for the isolation of bacterial and plant EVs (exosomes, microvesicles, and apoptotic bodies in plants). In terms of EV quantities, the analysis indicated total mean peribacteroid vesicle counts of 4.5 × 10^11^, 4.97 × 10^10^, 1.80 × 10^10^, 4.33 × 10^10^ particles·g of nodule^–1^ from the symbiotic pairs of soybean-HH103, bean-CIAT 899, and *L. burttii*-HH103/-CIAT 899, respectively (Figure 1E–H). These results underscore intensive EV transport across this interkingdom interface in all examined symbiotic partnerships. Indeed, the literature highlights the presence of proteins from both eukaryotic and prokaryotic symbionts in this intimate space, emphasizing its significance as a crucial, often underestimated component for symbiotic nitrogen fixation.^45−48^

EV yields were likewise quantified via fluorescence detection using the FM1–43 fluorophore (Figure 1I,J). By this approach overall lipid content is quantified. It was observed that there is a higher fluorescence intensity in EVs obtained from the symbiotic pairs bean-CIAT 899 and soybean-HH103 (Figure 1I) than in the pairs of *L. burttii*-CIAT 899 and *L. burttii-*HH103 (Figure 1J). Comparatively, the lipid dye measurements (Figure 1I,J) are in line with the scattering-light-based analysis by NanoSight (Figure 1E–H).

EVs from the peribacteroid space from all symbiotic pairs were further used for differential quantitative and qualitative analyses of their protein content by the BCA assay and SDS-PAGE, respectively. The average protein concentration of the samples from the symbiotic pairs of bean-CIAT 899 and soybean-HH103 was 964.0 ± 42.1 and 1873.4 ± 280.0 μg/mL, respectively **(**Figure 1K). However, the average protein concentration in the EVs of the symbiotic pairs of *L. burttii*-CIAT 899 and *L. burttii*-HH103 was 199.3 ± 126.6 and 147.9 ± 23.6 μg/mL, respectively (Figure 1L). To compare EV-encased protein patterns, all samples were loaded at the same concentration onto SDS gels and stained with silver staining (Figure 1M,N). The silver staining gels displayed differential EV-contained protein patterns among the symbiotic partners. These findings point out a specialized adapted protein cargo by the peribacteroid EVs that depends on which rhizobium and legume are engaged.

### Scarce Number of Proteins are Present in Peribacteroid EVs from the Different Rhizobia–Legume Combinations

**Figure 2:**
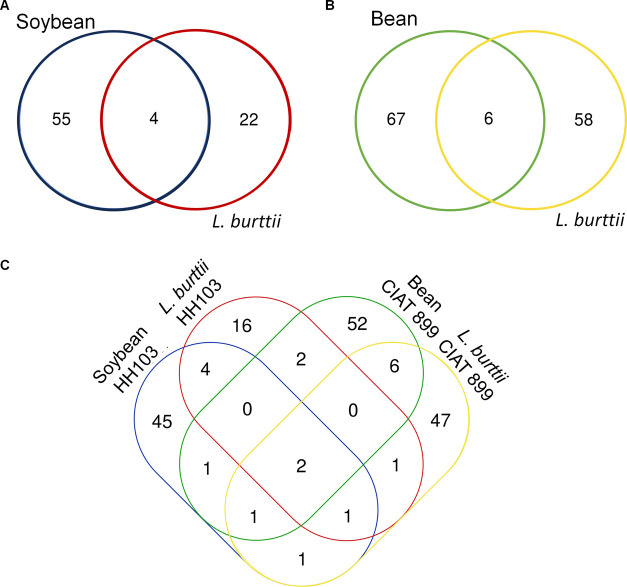
Venn diagram showing the protein overlap between the four different proteomic samples based on protein SeqNames (A, B) and the B2Go protein description (C).

Peribacteroid EVs from *G. max* and *L. burttii* nodules infected by *S. fredii* HH103 were evaluated by proteome profiling through liquid chromatography/tandem mass spectrometry (LC-MS/MS) identification. We identified 118 and 39 unique peptides corresponding to 59 and 26 different bacterial proteins from soybean and *L. burttii* peribacteroid spaces, respectively (Figure 2, Supporting Tables S1, S2, and S7). Among these proteins, only 4 were detected in the symbiosome EVs of both eukaryotic hosts (Table 1). We found an enzyme responsible for lipid A biosynthesis, lauroyl acyltransferase (CCE96230.1). This enzyme is involved in the incorporation of very long-chain fatty acids (VLCFAs) into lipid A of the LPS within bacteroids, apparently by EV trafficking. Notably, previous research has reported a higher proportion of VLCFAs in lipid A derived from nodules in comparison to that of free-living bacteria. Furthermore, the absence of these fatty acids in the LPS of microsymbionts has been observed to influence the structure and morphology of bacteroids.^49,50^ The impact of VLCFAs on rhizobial abilities to counteract the plant defense system and efficiently infect it has been hypothesized, although this has not been conclusively validated yet. However, it has been evidenced that while these bacteria might not necessarily require VLCFAs for survival within nodules, they do rely on these compounds to enhance their overall adaptation and thrive within this environment, rendering their existence significantly more conducive.^51^ Another interesting protein found in peribacteroid EVs from HH103 when nodulating with both host plants is F0F1 synthase subunit β (CCE97634.1), which is upregulated during the symbiotic interaction between soybean- and bean-compatible rhizobia.^52,53^ Since this subtype of prokaryotic ATPase/synthase holds pivotal importance in the energy metabolism of bacteroids, its presence within the EVs might indeed be a consequence of its substantial production within these nitrogen-fixing factories. Thus, the overproduction of this enzyme within the bacteroids might presumably result in its encapsulation into the EVs. This might facilitate its exchange within the bacterial cells of the nodule, potentially enhancing ATP generation and underpinning energy-consuming processes vital for nitrogen fixation within the symbiotic system.

**Table 1:** Common Proteins between Different Peribacteroid Space Isolated EVs of *S. fredii* HH103 and *R. tropici* CIAT 899 According to Their B2Go Protein Description^a^

SeqName	B2Go description	GO annotations
*S. fredii* HH103 shared proteins detected in EVs isolated from soybean and *L. burttii*		
CCE96230.1	lipid A biosynthesis lauroyl acyltransferase	C: plasma membrane; P: glycolipid biosynthetic process; F: acyltransferase activity
CCE97634.1	F0F1 ATP synthase subunit β	F: ATP binding; C: plasma membrane; P: proton motive force-driven ATP synthesis; F: ATP hydrolysis activity; C: proton-transporting ATP synthase complex, catalytic core F(1); F:proton-transporting ATP synthase activity, rotational mechanism; F: proton-transporting
CCE99650.1	DNA ligase D	F: DNA binding; F: DNA-directed DNA polymerase activity; F: DNA ligase (ATP) activity; F: exonuclease activity; F: ATP binding; P: DNA repair; P: DNA recombination; P: DNA biosynthetic process
CCF00065.1	CmpA/NrtA family ABC transporter substrate-binding protein	
CCE96218.1/CCE96988.1	PAS domain S-box protein	F: phosphorelay sensor kinase activity; P: phosphorelay signal transduction system; F: calmodulin-dependent protein kinase activity; F: ATP binding; P: regulation of DNA-templated transcription; P: blue light signaling pathway; F: blue light photoreceptor activity; P: phosphorylation; F: protein serine kinase activity
*R. tropici* CIAT 899 shared proteins detected in EVs isolated from bean and *L. burttii*		
AGB72165.1	cell cycle two-component system response regulator CtrA	P: phosphorelay signal transduction system; F: DNA binding; P: regulation of DNA-templated transcription
AGB72332.1	transketolase	F: transketolase activity; F: metal ion binding
AGB72959.1	helicase-related protein	F: RNA helicase activity; F: hydrolase activity
AGB73355.1	ABC transporter ATP-binding protein	F: ATP binding; F: ATP hydrolysis activity
AGB71388.1	ABC-type transporter Mla subunit MlaD	C: extracellular region; P: lipid transport; F: lipid binding; C: membrane; P: lipoprotein metabolic process
AGB75654.1	sarcosine oxidase subunit α family protein	F: sarcosine oxidase activity; P: tetrahydrofolate metabolic process
AGB72925.1/AGB70113.1	sn-glycerol-3-phosphate ABC transporter ATP-binding protein UgpC	F: ATP binding; P: carbohydrate transport; F: ATP hydrolysis activity; C: ATP-binding cassette (ABC) transporter complex; P: transmembrane transport; F: ABC-type transporter activity
AGB71395.1/AGB71699.1/AGB74627.1	ABC transporter substrate-binding protein	C: outer membrane-bounded periplasmic space; C: ATP-binding cassette (ABC) transporter complex; P: transmembrane transport
AGB69911.1/AGB74672.1	methyl-accepting chemotaxis protein	F: transmembrane signaling receptor activity; P: chemotaxis; P: signal transduction; C: membrane; F: oxygen binding; F: heme binding
*S. fredii* HH103 and *R. tropici* CIAT 899 shared proteins detected in EVs isolated from soybean, bean and *L. burttii*		
CCE95810.1	ABC transporter ATP-binding protein	F: ATP binding; C: membrane; F: ATP hydrolysis activity; P: transmembrane transport; F: ABC-type transporter activity
CCE97944.1	ABC transporter substrate-binding protein	P: carbohydrate transport; C: periplasmic space

a C: cellular component; P: biological process; F: molecular function.

LC-MS/MS analysis of the *R. tropici* CIAT 899 revealed the presence of 134 and 114 unique peptides corresponding to 73 and 64 different bacterial proteins from bean and *L. burttii* peribacteroid EVs, respectively (Figure 2, Supporting Tables S3, S4, and S8). Among the proteins found in the symbiosome EVs, only 6 were common between bean and *L. burttii* vesicles (Table 1), including the cell cycle two-component system response regulator CtrA (AGB72165.1). It has been reported that depletion of CtrA in *S. meliloti* induces bacterial cell swelling and genome endoreduplication and increases the permeability of the cell membrane, which in turn causes bacteroid terminal differentiation inside of indeterminate nodules of legumes belonging to the inverting-repeat lacking clade (IRLC) such as Medicago.^54^ However, in the determinate nodules of *P. vulgaris* and *L. burttii*, rhizobia do not undergo repeated DNA replication without cytokinesis, which makes bacteroids comparable to free-living bacteria regarding their genomic DNA content, cell size, and viability.^55^ Unfortunately, among rhizobia, “the CtrA Master Regulator of the cell cycle” has been only studied in *S. meliloti* so far.^56^ This rhizobial species presents an extremely narrow host range that only includes a few IRLC legumes. Thus, *S. meliloti* symbiosomes contain only one bacteroid that undergoes endoreplication and terminal differentiation. Because of this, it is difficult to extrapolate the current knowledge of the role of the *S. meliloti* CtrA protein to other rhizobia that nodulate non-IRLC legumes, such as *R. tropici*. In any case, the presence and interbacterial exchange of CtrA along the bacteroids by means of EVs might serve as a resort to ensure bacteroid division and their viability inside host cells. This transportation mechanism might be essential for coordinating and sustaining bacteroid proliferation and viability during the symbiotic interaction with bean and *L. burttii*.

Another protein found in EVs of the peribacteroid space from both plants infected with CIAT 899 was a transketolase (AGB72332.1), a key enzyme for a bacterial nonoxidative pentose phosphate pathway. This enzyme plays a central role in modulating the carbon flow necessary for a successful symbiosis in *Sinorhizobium meliloti*.^57^ The presence of a transketolase in the lumen of bacteroid EVs could be just a consequence of its boosted production within nitrogen-fixing cells. Alternatively, its EV-mediated trafficking across bacterial cells could potentially support and coordinate essential metabolic functions in the overall microsymbiont population. Finally, two proteins related to ABC-type transport were also found in the peribacteroid EVs of CIAT 899 isolated from both plants. This fact could be attributable to the described roles of this kind of transporter during the symbiosis. One of them is an ATP-binding cassette-type ABC transporter (AGB73355.1). Along these lines, it has been described that a soybean ATP-binding cassette-type transporter is responsible for the plant exudation of genistein, a flavonoid that induces the activation of nodulation-relevant genes in rhizobia.^58^ One possibility might be that the delivery of the bacterial transporter mentioned above into target legume cells via EV transmission could enhance the secretion of specialized flavonoids, thereby promoting the rapid development of the nitrogen-fixing nodule to the benefit of both symbiotic partners. Similarly, these ATP-dependent transporters appear to carry active cytokinins from plants, crucial for the successful formation of nitrogen-processing nodules.^59^

The ABC-type transporter Mla subunit MlaD (AGB71388.1) was likewise found in peribacteroid EVs from *L. burttii* and *P. vulgaris* nodules colonized by CIAT 899. MlaD is one of the elements of the Mla system (maintenance of outer membrane lipid asymmetry), which is necessary for trafficking diacylated phospholipids across the periplasm. Specifically, MlaD is an inner membrane-residing protein that binds and transfers phospholipids from the inner to the outer membrane.^60^

To ease a comparative analysis between the two rhizobial strains, despite the different SeqName annotation (accession numbers) of bacterial proteins, we used B2Go software analysis, which assigns a standardized protein description according to Blast. However, relying solely on the Blast2Go annotation to infer the presence of homologous proteins between different species can lead to errors as some proteins may not meet coverage and identity criteria. Therefore, we verified their coverages, overlaps, and sizes by performing a Blastp with the selected proteins. Two ABC-type transporters were consistently found in the EVs isolated from the four peribacteroid spaces inspected (CCE97944.1/AGB74678.1, CCE95810.1/AGB74737.1; Figure 2, Table 1). These transporters are critical for bacterial adaptation, with roles in cellular physiology, including the uptake of nutrients, exclusion of cellular residues, energy generation, and cellular signaling, among others. One of the two pairs of ABC-type transporters identified could be considered as orthologs since they display a certain degree of homology (CCE95810.1/AGB74737.1; 41% of coverage, 29,63% of identity). This pair of transporters shares amino acid homology with an ABC transporter ATP-binding protein that uptakes proline betaine, an osmoprotectant, to overcome the osmotic stress during different stages of the symbiotic process.^61,62^ Interbacterial exchange of this compatible solute transporter via EVs might thus be beneficial to endure ever-changing osmotic insults. The finding of this transporter in all analyzed peribacteroid EVs suggests widespread adaptation of rhizobia to the osmotic perturbances within the nodule. Furthermore, this approach revealed a new subset of proteins with the same Blast2Go description, an additional pair of proteins by HH103 host plants, and four extra pairs by CIAT 899 ones (Figure 2, Table 1). Among them, we highlight a set of PAS domain S-box proteins for HH103 (CCE96218.1 for soybean and CCE94881.1 for *L. burttii*). These proteins are bacterial inner sensors of oxygen tensions and redox potential through a histidine kinase signal transduction pathway.^63^ Thus, these proteins could potentially monitor oxygen concentrations within bacteroid cells in the peribacteroid space and may contribute to ensuring the activity of the O_2_-sensitive nitrogenase, along with leghemoglobins.^64^ However, despite the functioning of both versions of PAS domain S-box proteins being related or similar, the Blastp comparison demonstrated that these proteins are not homologous. In the case of CIAT 899, we highlight a pair of homologous methyl-accepting chemotaxis proteins (AGB69911.1 for bean and AGB74672.1 for *L. burttii*; 40% of coverage, 51,57% of identity), which are repressed during nodulation to abolish the bacteroid chemotaxis.^65^ In this case, EV secretion might be the means to dispose of such proteins to avoid unwanted chemotaxis during nodule formation. Additionally, two similar ABC transporter ATP-binding proteins of the sn-glycerol-3-phosphate (AGB72925.1 for bean and AGB70113.1 for *L. burttii*; 98% of coverage, 53.93% of identity), which is an essential intermediate in the biosynthesis of phospholipids,^66^ were likewise detected. In this scenario, EVs may mediate phospholipid recycling. The secretion of essential membrane-residing biological components via symbiosome EVs further emphasizes a notably active production and dynamic exchange of these elements across the peribacteroid space. Ultimately, bacteroids and plants can capitalize on the biological functions of these components by their reacquisition into each cell′s lumen.

### Protein Profile of Peribacteroid EVs is Adapted to the Host Legume

According to the B2Go description, most of the proteins encountered in EVs isolated from *S. fredii* HH103 and *R. tropici* CIAT 899 peribacteroids developed within their respective host plants were exclusive to each symbiotic partner (Supporting Tables S1–S4). This observation suggests that rhizobia species evolved a host-dependent mechanism to differentially load EVs in an adaptation to each specific symbiosis. Overall, 52 out of 59 and 19 out of 29 proteins were specifically identified in HH103 bacteroid-derived EVs in soybean and *L. burttii* plants, respectively. In the case of CIAT 899, 61 out of 73 unique proteins for bean and 53 out of 64 for *L. burttii* were detected in the EV isolates from peribacteroid spaces in both plant nodules.

HH103 nodulation with its natural host, the soybean, resulted in the identification of a large variety of EV-encased proteins (Table 2). Among them, the FixI protein (accession no. CCE96218.1) was found. This protein is an oxidase that is required for microaerobic bacteroid respiration.^67^ Its coding gene is comprised within the *fixGHIS* operon, which is located downstream of the *fixNOQP* operon. Both operons are responsible for the assembly of the symbiotically essential *cbb3*-type heme-copper oxidase complex, whose deletion results in defective symbiotic nitrogen fixation and a reduction of the cytochrome oxidase activity. In particular, FixI seems to be a copper-uptake ATPase due to its high similarity to the CopA protein of *Enterococcus hirae*. Consequently, this protein may participate in the uptake and metabolism of copper required for the assembly of the dinuclear center of cytochrome *cbb3* oxidase,^68^ which is essential for nitrogen fixation in symbiosomes.^69^ Another protein that drew our attention was the monocarboxylate transporter 12 MCT (acquisition number CCE95278.1). It has been discovered that in *Rhizobium leguminosarum* MCT transports alanine and other monocarboxylates, including pyruvate and lactate.^70^ Alanine can make up to 26% of the total nitrogen secreted by bacteroids of *R. leguminosarum* isolated from pea nodules.^71^ Certain studies have reported that in the case of soybean bacteroids, alanine is the exclusive product of nitrogen fixation.^72^ Therefore, this transporter might be carrying nitrogen into the plant in the form of alanine through EVs secreted by bacteroids. Additionally, the EV-detected polyamine ABC transporter (accession number CCE95810.1) might orchestrate crucial roles in the symbiotic context. Polyamines (PAs) are aliphatic amines that play vital roles in numerous physiological processes, encompassing responses to abiotic stresses and interactions with microbes and are recognized as key regulators of plant growth, development, and responses to stress.^73^ The importance of PA transport during stress endurance is noteworthy, especially considering that the overexpression of various related genes in rhizobia has already proven to have positive effects on symbiosis. This is due to the fact that rhizobia must be able to cope with multiple stress conditions during the infection course of action and once inside the nodule in order to culminate with effective symbiosis establishment.^74^ Besides, PAs enable the plant to nodulate efficiently as broadly evidenced.^75−78^ Therefore, PA affinity and sequestration by EV-encapsulated transporters might be sustaining the exchange of this molecule interbacterial and interkingdom.

**Table 2:** Selected *S. fredii* HH103 Proteins Identified in the EVs Present in the Peribacteroid Spaces from the Two Different Host Plants

SeqName	B2Go description	host plant	putative role in symbiosis	references
CCE96218.1	cation-translocating P-type ATPase	soybean	responsible for the symbiotically essential *cbb3*-type heme-copper oxidase complex	Preisig et al., 1996
CCE95278.1	MFS transporter	soybean	in *R. leguminosarum* transports alanine and other monocarboxylates to the plant	Hosie et al., 2002
CCE96562.1	ABC transporter ATP-binding protein	soybean	transports polyamines that are involved in the nodulation ability of the plant	Hidalgo-Castellanos et al., 2019
CCE98405.1	glutathione synthase	*L. burttii*	in *S. meliloti* is required for optimal nodulation	Harrison et al., 2005
CCE98274.1	AEC family transporter	*L. burttii*	it could be helping in the uptake of auxin to the plants	Ung et al., 2022
CCE97330.1	sulfite exporter TauE/SafE family protein	*L. burttii*	in the symbiosomal membrane of *L. japonicus*, a sulfate transporter has been found to be specifically and highly expressed.	Wienkoop and Sallbach, 2003

Regarding the proteins found in the EVs of HH103 when nodulating with *L. burttii*, the glutathione synthetase (GSH) protein might have relevant functions in symbiosis (accession number CCE98405.1). Glutathione (GSH) serves as a multifaceted antioxidant capable of quickly neutralizing reactive oxygen species (ROS). Beyond its antioxidant role, GSH is pivotal to numerous essential functions in plants, including sulfur transport and storage, protein and DNA synthesis, resilience to both abiotic and biotic stressors, as well as detoxification of xenobiotics, air pollutants, and heavy metals.^79,80^ There is an active ascorbate-GSH cycle occurring within the root nodules, which relies on a steady GSH supply. This cycle is crucial to safeguard nitrogen fixation from ROS.^81^ Additionally, various studies nourish the notion that the production of GSH by plants and bacteria is a fundamental aspect of initiating and sustaining symbiosis.^82^ Mutant strains of *S. meliloti* defective in GSH biosynthesis exhibited reduced symbiotic traits, which highlights the prominence of GSH throughout this process.^83^ Encapsulating this enzyme inside the EVs might endow them with glutathione affinity and/or production capacities, promoting its encasement in the vessels. Thus, this compound could be steadily secreted into the peribacteroid space to support the chemical reactions underlying nitrogen fixation and preserve this pathway from damage caused by ROS.

Furthermore, a putative auxin efflux carrier (AtPIN8; accession number CCE98274.1) was found in EVs released by HH103 bacteroids in *L. burttii*. This protein is an auxin transporter typically codified by eukaryotic genes that structurally and homology-wise resembles that of the protein PIN8 (accession number AT5G15100), a transporter from *Arabidopsis thaliana*. This protein is an elevator-type transporter that controls auxin export from the cytosol to the extracellular space.^84^ Auxins are closely involved in cell cycle control, differentiation of vascular tissues (development of lateral roots and nodules), and the formation of the infection thread,^85^ all relevant aspects to symbiosis. Hence, this transporter might be incorporated in the EVs aiming to recruit auxins that can ultimately be taken up by the plant cells. Finally, the sulfite exporter protein SafE (accession number CCE97330.1) was found in HH103 EVs from *L. burttii* nodules. Sulfur is an indispensable nutrient for plants as it constitutes the amino acids cysteine (Cys) and methionine (Met), as well as metal cofactors, coenzymes, and secondary metabolites.^86^ Nodulated legumes display a heavy reliance on sulfur: those supplied with abundant sulfur have increased rates of N_2_ fixation, while those cultivated in sulfur-limited soils show decreased nitrogenase activity.^87^ Sulfur is taken up as sulfate by plant cells through sulfate transporters and needs to be reduced to organic sulfide, which is reduced to sulfite in the first place. A sulfate transporter in the symbiosomal membrane of *Lotus japonicus* has been identified. This transporter was found to be specifically and highly expressed in the symbiosomal membrane, suggesting an important active sulfate transport in symbiosis.^88^ Hence, EV-residing SafE might emerge as a sulfite carrier to release it into adjacent plant cells to incorporate into its sulfur metabolism by its reduction to organic sulfide.

Among the identified proteins in CIAT 899 during its nodulation with its natural host, the bean, different RND (Resistance Nodulation and Cell Division) efflux pump proteins predominate (Table 3). This family of membrane transporters is involved in resistance to heavy metals (R), nodulation (N), or cell division (D), hence the acronym RND.^89,90^ One of the detected proteins corresponds to that encoded by the *nolG* gene of CIAT 899 (accession number AGB73763.1). Furthermore, the *nolG* gene of *S. meliloti* is activated in response to the inducing flavonoid luteolin and is required for optimal nodulation in alfalfa.^91^ Despite numerous efforts to unravel the functional traits of NolG and the transport system to which it belongs, the specific function of this protein remains unclear.

**Table 3:** Selected *R. tropici* CIAT 899 Proteins Identified in the EVs Present in the Peribacteroid Spaces from the Two Different Host Plants

SeqName	B2Go description	host plant	putative role in symbiosis	references
AGB73763.1	efflux RND transporter permease subunit	bean	in *S. meliloti* is required for optimal nodulation with alfalfa.	Baev et al., 1991
AGB73463.1	carbamoyltransferase HypF	bean	in *M. huakuii* is essential in the nitrogen fixation process	Long et al., 2023
AGB72042.1	DEAD/DEAH-box helicase	bean	involved in some aspects of RNA trafficking between rhizobia and legume	Cai et al., 2019; He et al., 2021
WP_041678072.1	DEAD/DEAH-box helicase	*L. burttii*	involved in some aspects of RNA trafficking between rhizobia and legume	Cai et al., 2019; He et al., 2021
AGB70855.1 and AGB69578.1	penicillin-binding proteins (PBP)	*L. burttii*	*Bradyrhizobium* strains ORS278 and ORS285 have a DD-carboxypeptidase enzyme (DD-CPase1) belonging to the PBP family, which is expressed during symbiosis	Gully et al., 2016

Another protein identified in CIAT 899 EVs was the hydrogenase maturation factor HypF (AGB73463.1). A recent study has revealed that the hydrogenase maturation protein HypE of the symbiont *Mesorhizobium huakuii* is essential during the nitrogen fixation route.^92^ This protein was also related to other crucial bacterial roles, such as energy and electron provision, ROS and pH-dependent detoxification, or differentiation into bacteroids and nodule senescence.^92^ Given that its associated biological roles are rather generic, its function within the EVs, if any, remains uncertain.

In addition to various proteins involved in defense and stress response, it is remarkable that 10% of the proteins identified in the EVs were related to RNA metabolism. An example was the protein AGB72042.1, which corresponds to an RNA helicase of the DEAD/DEAH-box helicase family (Table 3). This family of helicases is involved in many aspects of RNA metabolism, including mRNA (mRNA) maturation, small RNA (sRNA) processing, or RNA transcription.^93^ In the case of cargo proteins of EVs isolated from *L. burttii* nodules, as well as in *P. vulgaris*, another DEAD/DEAH-box helicase family protein was identified (WP_041678072.1). Regarding small noncoding RNAs (sRNAs), it has been demonstrated that these molecules can travel from one organism to another to induce gene silencing in other cells, a phenomenon known as interkingdom RNA interference communication.^94^ Specifically, these sRNAs are known immunomodulators of the immune response and virulence processes in plants. For instance, in the plant–fungus warfare, sRNAs from pathogenic fungi navigate toward the cells of their host plant to silence the plant’s immune response. In turn, plant sRNAs are conveyed to the fungus to repress the pathogen’s virulence system.^95^ Importantly, it has been determined that plants release these sRNAs by means of EVs. RNA-binding proteins are the agents responsible for specifically loading these sRNAs into plant EVs.^28,96^

Regarding the role of sRNAs in the rhizobium–legume symbiosis, recent literature pinpoints the regulatory function of sRNAs synthesized by plants during symbiosis establishment.^97,98^ Conversely, research on RNA regulation by rhizobia is very limited, and so far, these studies have focused almost exclusively on *S. meliloti* symbiotic species. In this context, it was postulated that a large number of sRNAs are able to modulate the nodulation process. Nevertheless, very scarce aspects of the biosynthesis mechanism and function of these sRNAs have been disclosed.^99−101^ Some examples are the NfeR1 sRNA of *S. meliloti*, whose expression levels are highest under nitrogen starvation conditions and in bacteroids,^102,103^ or the 25 tRNA-derived small RNA fragments of *Bradyrhizobium japonicum*, which are signal molecules regulating soybean nodulation.^104^ Furthermore, it has been speculated that these sRNAs could concurrently target multiple genes in the plant genome. In line with all of the current knowledge on the interkingdom RNA interference field, it is arguably plausible that the identified family of RNA-binding proteins found in our rhizobial EVs are responsible for the differential encasement of specific sRNAs. This mechanism would enable rhizobial species to transfer tailored RNAs into the plant cell to modulate targeted genetic traits that lead to the enhancement of the symbiosis process. To challenge this hypothesis, a comparative transcriptomic study of EVs isolated from *P. vulgaris* bacteroids colonized by a CIAT 899 wild type and the RNA helicase gene-lacking strain could be carried out. By these means, ribonucleic acids specifically loaded into the EVs by the action of this helicase might be identified.

In addition, two penicillin-binding proteins (PBP) (AGB70855.1 and AGB69578.1) were detected in the EVs isolated from *L. burttii* nodules infected with CIAT 899. The PBP family proteins are among the most prominent peptidoglycan-modifying enzymes that play a role in the maintenance of cell shape, and they are involved in peptidoglycan biosynthesis. Curiously, *Bradyrhizobium* strains ORS278 and ORS285 have a DD-carboxypeptidase enzyme (DD-CPase1) belonging to the PBP family, which is expressed during symbiosis. The absence of this protein in *Bradyrhizobium* strains induced malformed and hypertrophied bacteroids in symbiosis with *Aeschynomene* plants. However, DD-CPase1 is dispensable for free-living growth or in symbiosis with the host plant, soybean.^105^ EVs might be supplied with PBPs to neighboring cells in order to maintain cell wall homeostasis. Thus, these results hint at the importance of the peptidoglycan layer in nodule development.

Noteworthily, among the proteins identified in the EVs isolated from the plant nodules colonized by CIAT 899 and HH103 strains, several proteins with unknown functions were detected. In fact, some of these hypothetical proteins are encoded by genes located in the symbiotic plasmid of both strains, which harbor most of the genes implicated in symbiosis.^106,107^ Hence, future endeavors to elucidate the role of these uncharacterized proteins, hypothetically related to nitrogen fixation, should be addressed.

Finally, proteome analysis carried out from the peribacteroid membrane of *Lotus japonicus* root nodules exposed the presence of several membrane-embedded proteins involved in signaling. In particular, three matches were obtained encoding proteins homologous to a receptor protein kinase in *Arabidopsis*.^88^ These data may suggest that some of the proteins contained in the EVs secreted by CIAT 899 and HH103 bacteroids into the peribacteroid space could act as ligands for the receptors located in the peribacteroid membrane and, consequently, trigger a signaling pathway in the symbiotic cell.

### Host-Specific Protein Cargo from Different Bacteroid EVs Affect the Same Biological Processes, Molecular Functions, and Cellular Components

**Figure 3:**
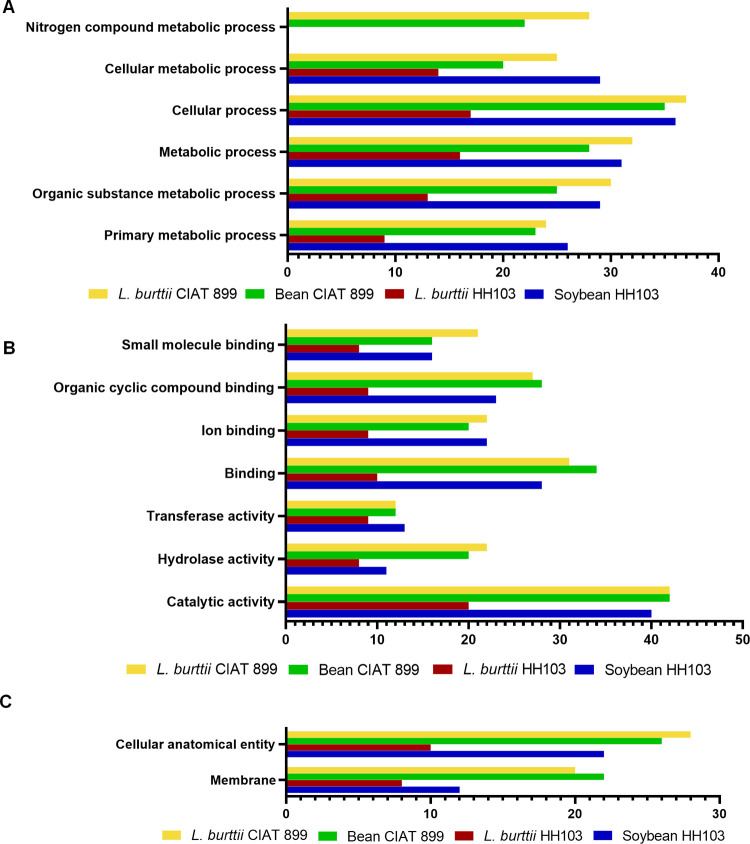
Peribacteroid EV proteins distributed according to gene ontology category enrichment and classified into biological process (A), molecular function (B), and cellular component (C). The most representative functional categories for enriched proteins from the four symbiotic pairs are shown. The *x-*axis represents the number of proteins belonging to the functional category indicated on the *y-*axis.

Based on gene ontology (GO) category enrichment, we then used detected proteins to carry out protein functional analysis using the UniProt Gene Ontology Annotation database integrated with Blast2Go software. The Blast2GO suite is a comprehensive bioinformatics tool designed for the functional annotation of sequences and data mining based on the GO vocabulary. It optimizes function transfer from homologous sequences using a sophisticated algorithm that considers factors such as similarity, homology extent, database selection, GO hierarchy, and quality of the original annotations. Blast2GO offers numerous features for visualization, management, and statistical analysis of annotation results, including gene set enrichment analysis. The tool supports InterPro, enzyme codes, KEGG pathways, GO direct acyclic graphs (DAGs), and GOSlim.^108^ Proteins were classified according to their functional characteristics into the biological process (BP) (Figures S1–S4), molecular function (MF) (Figures S5–S8), and cellular component (CC) (Figures S9–S12) categories. The most prominent functional categories of the protein cargo within EVs from HH103 bacteroids in both plants were almost identical. These categories include the primary metabolic process, cellular process, organic substance metabolic process, cellular metabolic process, and metabolic process (Figure 3A). The scrutiny of the MF of the identified proteins exposed a similar trend. In both soybean and *L. burttii*, the most abundant categories were the same: binding (especially ion, organic cyclic, and small molecules) and catalytic activity (hydrolase and transferase) (Figure 3B). Finally, the most represented cellular component (CC) categories from the protein cargo of bacteroid EVs were also identical between both HH103 host plants: the membrane and the cellular anatomical entity (Figure 3C).

An analogy was found when classifying the proteins identified during the symbiosis with bean and *L. burttii* by CIAT 899. The main functional BP, MF, and CC categories of the protein cargo found in EVs from peribacteroids of both hosts were similar to those obtained for HH103 when nodulating with their respective legumes and did not differ between legume hosts. The main BPs were the primary metabolic process, cellular process, organic substance metabolic process, metabolic process, and nitrogen compound metabolic process. The most overrepresented MF was also catalytic activity (hydrolase and transferase) and binding (ion and organic cyclic compound), while the most abundant categories of CC belonged to cellular anatomical entities and membranes. Collectively, these findings indicate that although proteins encapsulated in each rhizobial bacteroid EV are of a different nature, they target the same cellular processes, components, and reactions regardless of the legume host. Thus, despite rhizobia having evolved different molecular strategies to enhance the effectiveness of nodulation during the early stages of symbiosis, the molecular dialogue mediated by EVs merges when the rhizobium–legume relationship becomes more intimate.

## Conclusions

To date, comprehensive research dissecting the roles of EVs in one of the most complex naturally occurring molecular dialogues, rhizobium–legume symbiosis, has been neglected. During the different stages of the symbiotic process, rhizobia and their host plants establish a very specific and controlled intercellular trafficking of signal molecules. Thus, as conveyors of a broad range of molecules into the target cell, EVs are capturing attention in the field. Unprecedently, in this study, we devised a straightforward procedure to isolate EVs from bacteroids of legume nodules of different symbiotic partners. Implementation of this approach allowed the proteomically driven discovery of potential new actors involved in the symbiotic process. Additionally, we found that the biological processes, molecular functions, and cellular components mediated by EV are conserved independently of the symbiotic pair. The presented procedure opens the gate to the holistic characterization by multiomic techniques of EV-driven crosstalk between the plant host and the bacterium with unrestricted potential to develop green technologies for sustainable agriculture.

## Supporting Information Available

list of *Sinorhizobium fredii* HH10 proteins identified in the extracellular vesicles isolated from the peribacteroid space of soybean nodules infected with *Sinorhizobium fredii* HH103 (Table S1) (XLSX)
List of *Sinorhizobium fredii* HH103 proteins identified in the extracellular vesicles isolated from the peribacteroid space of *Lotus burttii* nodules infected with *Sinorhizobium fredii* HH103 (Table S2) (XLSX)
List of *Rhizobium tropici* CIAT 899 proteins identified in the extracellular vesicles isolated from the peribacteroid space of bean nodules infected with *Rhizobium tropici* CIAT 899 (Table S3) (XLSX)
List of *Rhizobium tropici* CIAT 899 proteins identified in the extracellular vesicles isolated from the peribacteroid space of *Lotus burttii* nodules infected with *Rhizobium tropici* CIAT 899 (Table S4) (XLSX)
Proteome data set with *Glycine max* proteins identified in the extracellular vesicles isolated from the peribacteroid space of nodules infected with *Sinorhizobium fredii* HH103 (Table S5) (XLSX)
Proteome data set with *Phaseolus vulgaris* proteins identified in the extracellular vesicles isolated from the peribacteroid space of nodules infected with *Rhizobium tropici* CIAT 899 (Table S6) (XLSX)
Proteome data set with *Sinorhizobium fredii* HH103 proteins identified in the extracellular vesicles isolated from the peribacteroid space of nodules of *Glycine max* and *Lotus burttii* (Table S7) (XLSX)
Proteome data set with *Rhizobium tropici* CIAT 899 proteins identified in the extracellular vesicles isolated from the peribacteroid space of nodules of *Phaseolus vulgaris* and *Lotus burttii* (Table S8) (XLSX)
Combined hierarchical graph displaying *Sinorhizobium fredii* HH103 proteins identified in the membrane vesicles isolated from the peribacteroid space of soybean nodules and distributed according to gene ontology category enrichment and classified into the biological process affected (Figure S1); combined hierarchical graph displaying *Sinorhizobium fredii* HH103 proteins identified in the membrane vesicles isolated from the peribacteroid space of *Lotus burttii* nodules and distributed according to gene ontology category enrichment and classified into the biological process affected (Figure S2); combined hierarchical graph displaying *Rhizobium tropici* CIAT 899 proteins identified in the membrane vesicles isolated from the peribacteroid space of bean nodules and distributed according to gene ontology category enrichment and classified into the biological process affected (Figure S3); combined hierarchical graph displaying *Rhizobium tropici* CIAT 899 proteins identified in the membrane vesicles isolated from the peribacteroid space of *Lotus burttii* nodules and distributed according to gene ontology category enrichment and classified into the biological process affected (Figure S4); combined hierarchical graph displaying *Sinorhizobium fredii* HH103 proteins identified in the membrane vesicles isolated from the peribacteroid space of soybean nodules and distributed according to gene ontology category enrichment and classified into the molecular function affected (Figure S5); combined hierarchical graph displaying *Sinorhizobium fredii* HH103 proteins identified in the membrane vesicles isolated from the peribacteroid space of *Lotus burttii* nodules and distributed according to gene ontology category enrichment and classified into the molecular function affected (Figure S6); combined hierarchical graph displaying *Rhizobium tropici* CIAT 899 proteins identified in the membrane vesicles isolated from the peribacteroid space of bean nodules and distributed according to gene ontology category enrichment and classified into the molecular function affected (Figure S7); combined hierarchical graph displaying *Rhizobium tropici* CIAT 899 proteins identified in the membrane vesicles isolated from the peribacteroid space of *Lotus burttii* nodules and distributed according to gene ontology category enrichment and classified into the molecular function affected (Figure S8); **c**ombined hierarchical graph displaying *Sinorhizobium fredii* HH103 proteins identified in the membrane vesicles isolated from the peribacteroid space of soybean nodules and distributed according to gene ontology category enrichment and classified into the cellular component affected (Figure S9); combined hierarchical graph displaying *Sinorhizobium fredii* HH103 proteins identified in the membrane vesicles isolated from the peribacteroid space of *Lotus burttii* nodules and distributed according to gene ontology category enrichment and classified into the cellular component affected (Figure S10); combined hierarchical graph displaying *Rhizobium tropici* CIAT 899 proteins identified in the membrane vesicles isolated from the peribacteroid space of bean nodules and distributed according to gene ontology category enrichment and classified into the cellular component affected (Figure S11); Combined hierarchical graph displaying *Rhizobium tropici* CIAT 899 proteins identified in the membrane vesicles isolated from the peribacteroid space of *Lotus burttii* nodules and distributed according to gene ontology category enrichment and classified into the cellular component affected (Figure S12) (PDF)

## Notes

Proteomic data are available via ProteomeXchange with identifier PXD056185.
