# Supplementary material for: Extracellular Vesicle-Driven Crosstalk between Legume Plants and Rhizobia: The Peribacteroid Space of Symbiosomes as a Protein Trafficking Interface
Source: J Proteome Res. 2024 Dec 12;24(1):94–110. doi: 10.1021/acs.jproteome.4c00444 (PMC11705226; doi:10.1021/acs.jproteome.4c00444)
Supplement: Supplementary file 9 — pr4c00444_si_009.pdf [file pr4c00444_si_009.pdf]

Supporting Information

Extracellular vesicle-driven crosstalk between legume plants and rhizobia: the peribacteroid space of symbiosomes as protein trafficking interface

Paula Ayala-García[1#], Irene Herrero-Gómez[1#], Irene Jiménez-Guerrero[1], Viktoria Otto[2], Natalia Moreno de Castro[1], Mathias Müsken[3], Lothar Jänsch[4], Marco van Ham[4], José-María Vinardell[1], Francisco Javier López-Baena[1], Francisco Javier Ollero[1], Francisco Pérez-Montaño[1*], José Manuel Borrero-de Acuña[1*]

Table of content

Figure S1. Combined hierarchical graph displaying *Sinorhizobium fredii* HH103 proteins identified in the membrane vesicles isolated from the peribacteroid space of soybean nodules and distributed according to gene ontology category enrichment and classified into the biological process affected.

Figure S2. Combined hierarchical graph displaying *Sinorhizobium fredii* HH103 proteins identified in the membrane vesicles isolated from the peribacteroid space of *Lotus burttii* nodules and distributed according to gene ontology category enrichment and classified into the biological process affected.

Figure S3. Combined hierarchical graph displaying *Rhizobium tropici* CIAT 899 proteins identified in the membrane vesicles isolated from the peribacteroid space of bean nodules and distributed according to gene ontology category enrichment and classified into the biological process affected.

Figure S4. Combined hierarchical graph displaying *Rhizobium tropici* CIAT 899 proteins identified in the membrane vesicles isolated from the peribacteroid space of *Lotus burttii*

nodules and distributed according to gene ontology category enrichment and classified into the biological process affected.

Figure S5. Combined hierarchical graph displaying *Sinorhizobium fredii* HH103 proteins identified in the membrane vesicles isolated from the peribacteroid space of soybean nodules and distributed according to gene ontology category enrichment and classified into the molecular function affected.

Figure S6. Combined hierarchical graph displaying *Sinorhizobium fredii* HH103 proteins identified in the membrane vesicles isolated from the peribacteroid space of *Lotus burttii* nodules and distributed according to gene ontology category enrichment and classified into the molecular function affected.

Figure S7. Combined hierarchical graph displaying *Rhizobium tropici* CIAT 899 proteins identified in the membrane vesicles isolated from the peribacteroid space of bean nodules and distributed according to gene ontology category enrichment and classified into the molecular function affected.

Figure S8. Combined hierarchical graph displaying *Rhizobium tropici* CIAT 899 proteins identified in the membrane vesicles isolated from the peribacteroid space of *Lotus burttii* nodules and distributed according to gene ontology category enrichment and classified into the molecular function affected.

Figure S9. Combined hierarchical graph displaying *Sinorhizobium fredii* HH103 proteins identified in the membrane vesicles isolated from the peribacteroid space of soybean nodules and distributed according to gene ontology category enrichment and classified into the cellular component affected.

Figure S10. Combined hierarchical graph displaying *Sinorhizobium fredii* HH103 proteins identified in the membrane vesicles isolated from the peribacteroid space of *Lotus burttii*

nodules and distributed according to gene ontology category enrichment and classified into the cellular component affected.

Figure S11. Combined hierarchical graph displaying *Rhizobium tropici* CIAT 899 proteins identified in the membrane vesicles isolated from the peribacteroid space of bean nodules and distributed according to gene ontology category enrichment and classified into the cellular component affected.

Figure S12. Combined hierarchical graph displaying *Rhizobium tropici* CIAT 899 proteins identified in the membrane vesicles isolated from the peribacteroid space of *Lotus burttii* nodules and distributed according to gene ontology category enrichment and classified into the cellular component affected.

Supporting Table S1. List of *Sinorhizobium fredii* HH10 proteins identified in the extracellular vesicles isolated from the peribacteroid space of soybean nodules infected with *Sinorhizobium fredii* HH103.

Supporting Table S2. List of *Sinorhizobium fredii* HH103 proteins identified in the extracellular vesicles isolated from the peribacteroid space of *Lotus burttii* nodules infected with *Sinorhizobium fredii* HH103.

Supporting Table S3. List of *Rhizobium tropici* CIAT 899 proteins identified in the extracellular vesicles isolated from the peribacteroid space of bean nodules infected with *Rhizobium tropici* CIAT 899.

Supporting Table S4. List of *Rhizobium tropici* CIAT 899 proteins identified in the extracellular vesicles isolated from the peribacteroid space of *Lotus burttii* nodules infected with *Rhizobium tropici* CIAT 899.

Supporting Table S5. Proteome dataset with *Glycine max* proteins identified in the extracellular vesicles isolated from the peribacteroid space of nodules infected with *Sinorhizobium fredii* HH103.

Supporting Table S6. Proteome dataset with *Phaseolus vulgaris* proteins identified in the extracellular vesicles isolated from the peribacteroid space of nodules infected with *Rhizobium tropici* CIAT 899.

Supporting Table S7. Proteome dataset with *Sinorhizobium fredii* HH103 proteins identified in the extracellular vesicles isolated from the peribacteroid space of nodules of *Glycine max* and *Lotus burttii*.

Supporting Table S8. Proteome dataset with *Rhizobium tropici* CIAT 899 proteins identified in the extracellular vesicles isolated from the peribacteroid space of nodules of *Phaseolus vulgaris* and *Lotus burttii*.

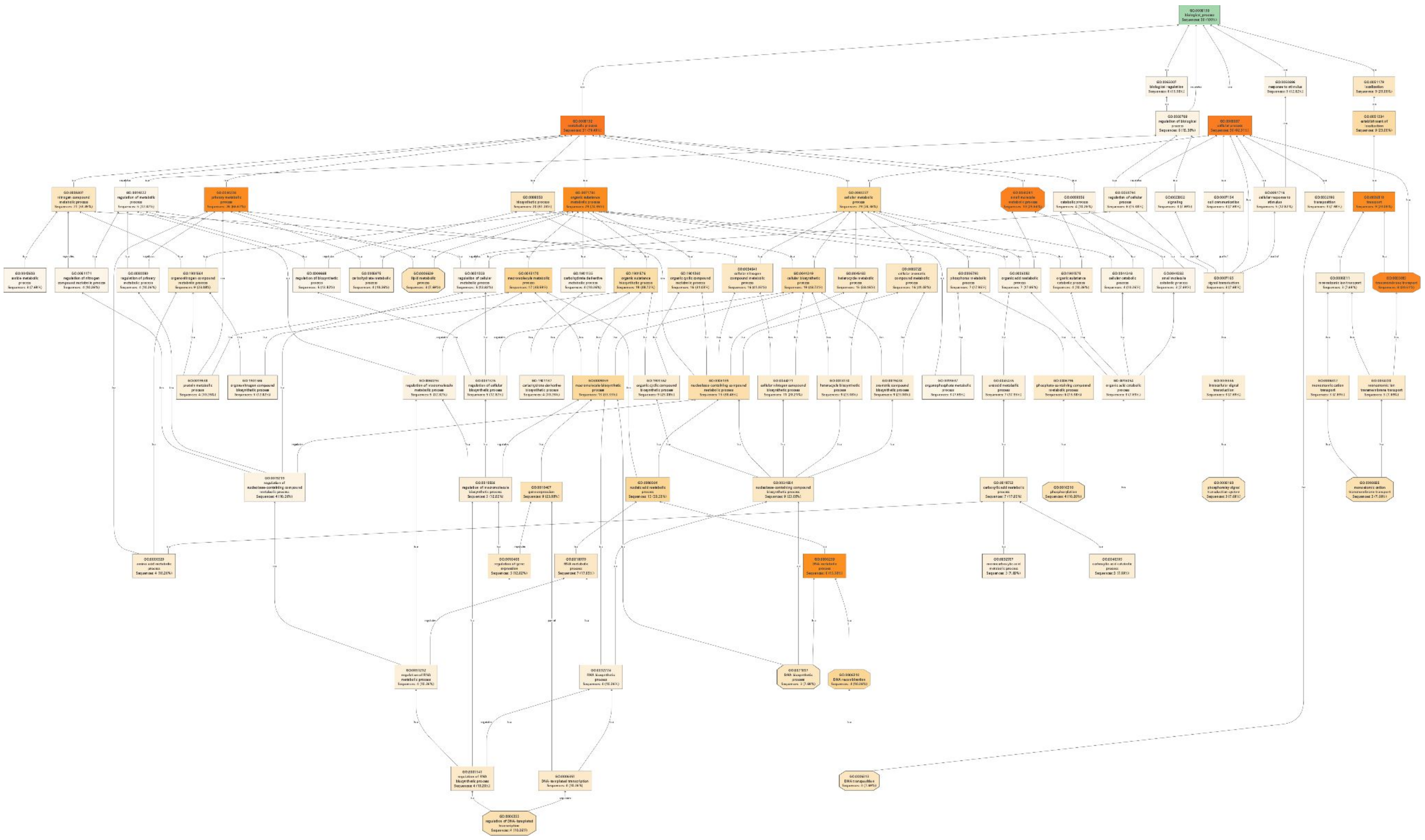

Figure S1. Combined hierarchical graph displaying *Sinorhizobium fredii* HH103 proteins identified in the membrane vesicles isolated from the peribacteroid space of soybean nodules and distributed according to gene ontology category enrichment and classified into the biological process affected. The boxes with darker shades represent the Blast2Go categories with higher representation according to the percentage of proteins encountered.

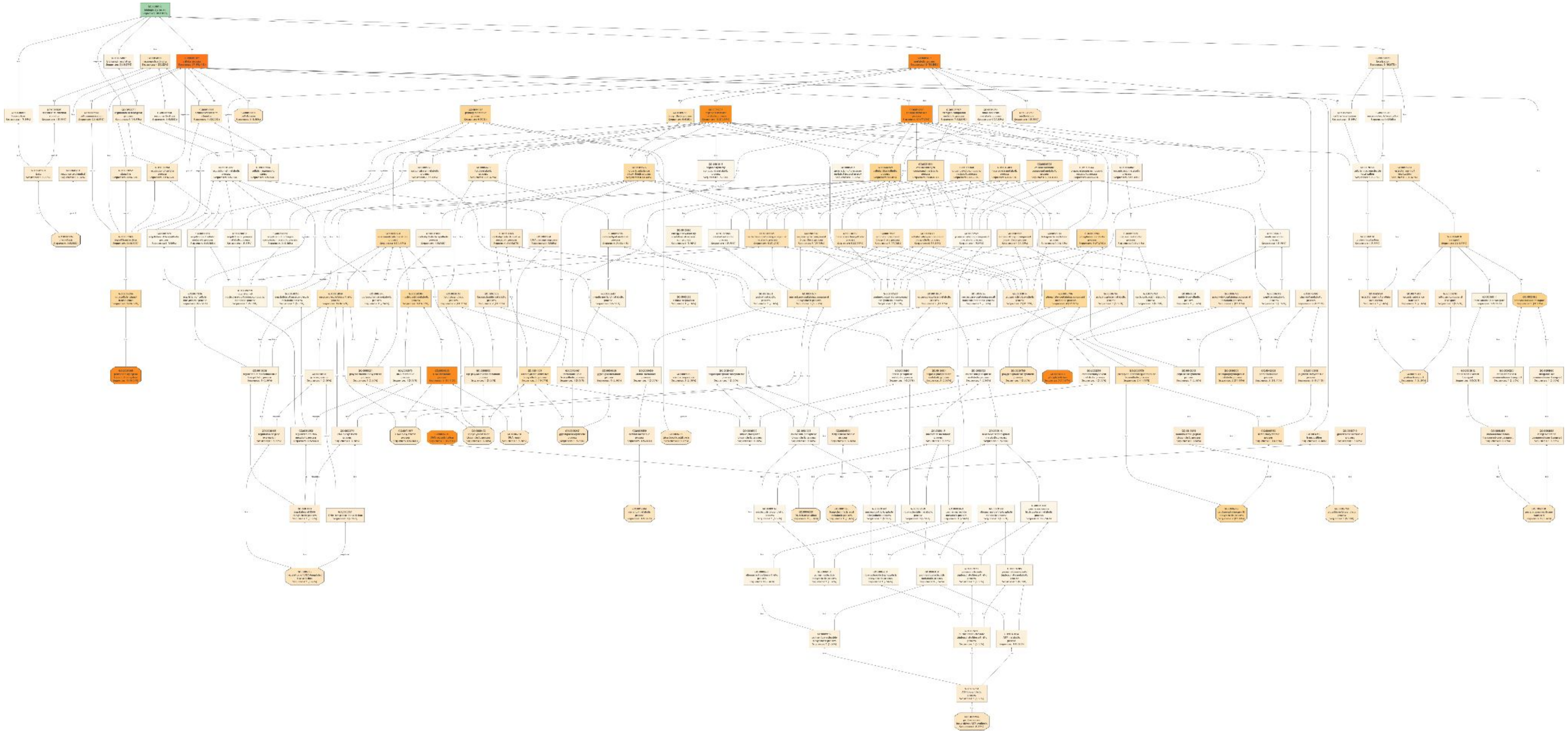

Figure S2. Combined hierarchical graph displaying *Sinorhizobium fredii* HH103 proteins identified in the membrane vesicles isolated from the peribacteroid space of *Lotus burttii* nodules and distributed according to gene ontology category enrichment and classified into the biological process affected. The boxes with darker shades represent the Blast2Go categories with higher representation according to the percentage of proteins encountered.

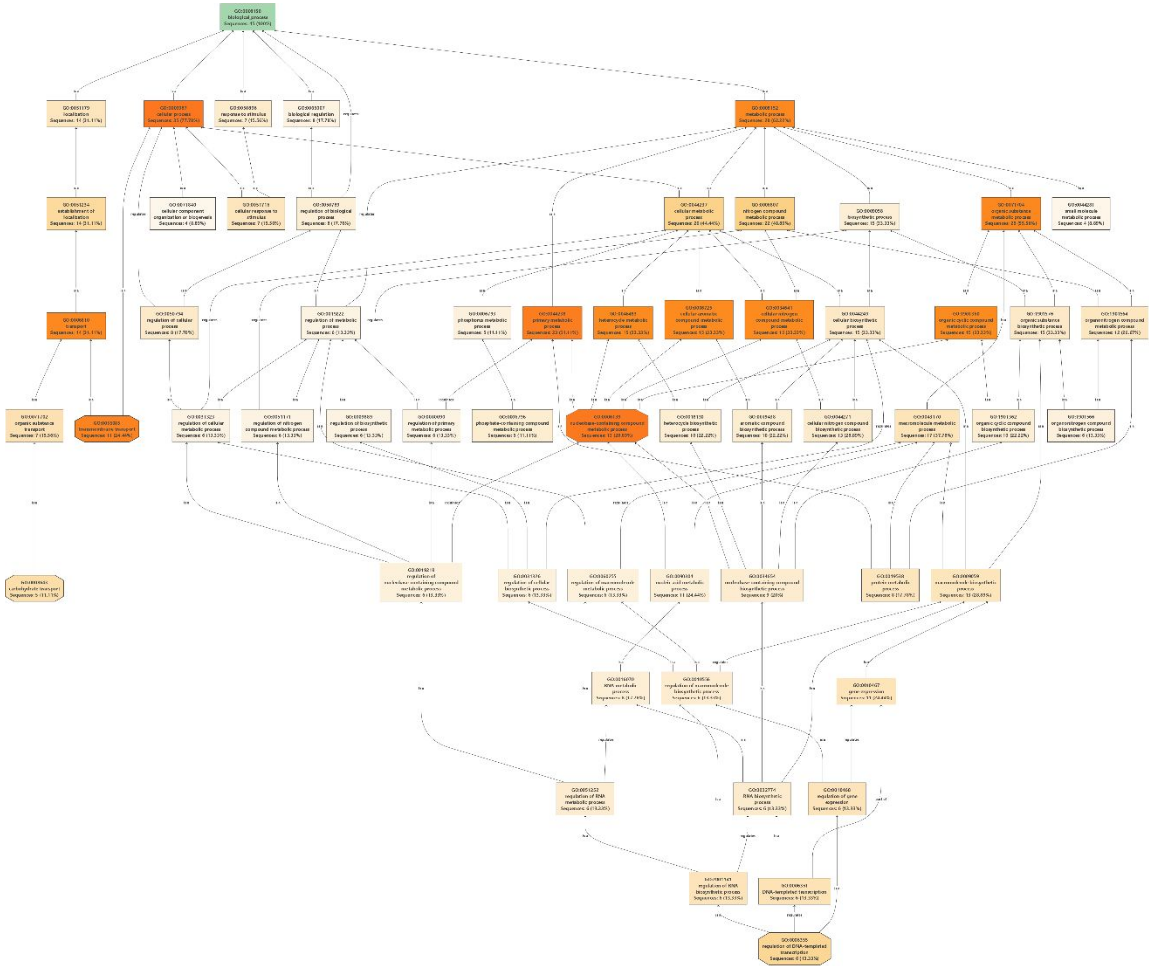

Figure S3. Combined hierarchical graph displaying *Rhizobium tropici* CIAT 899 proteins identified in the membrane vesicles isolated from the peribacteroid space of bean nodules and distributed according to gene ontology category enrichment and classified into the biological process affected. The boxes with darker shades represent the Blast2Go categories with higher representation according to the percentage of proteins encountered.

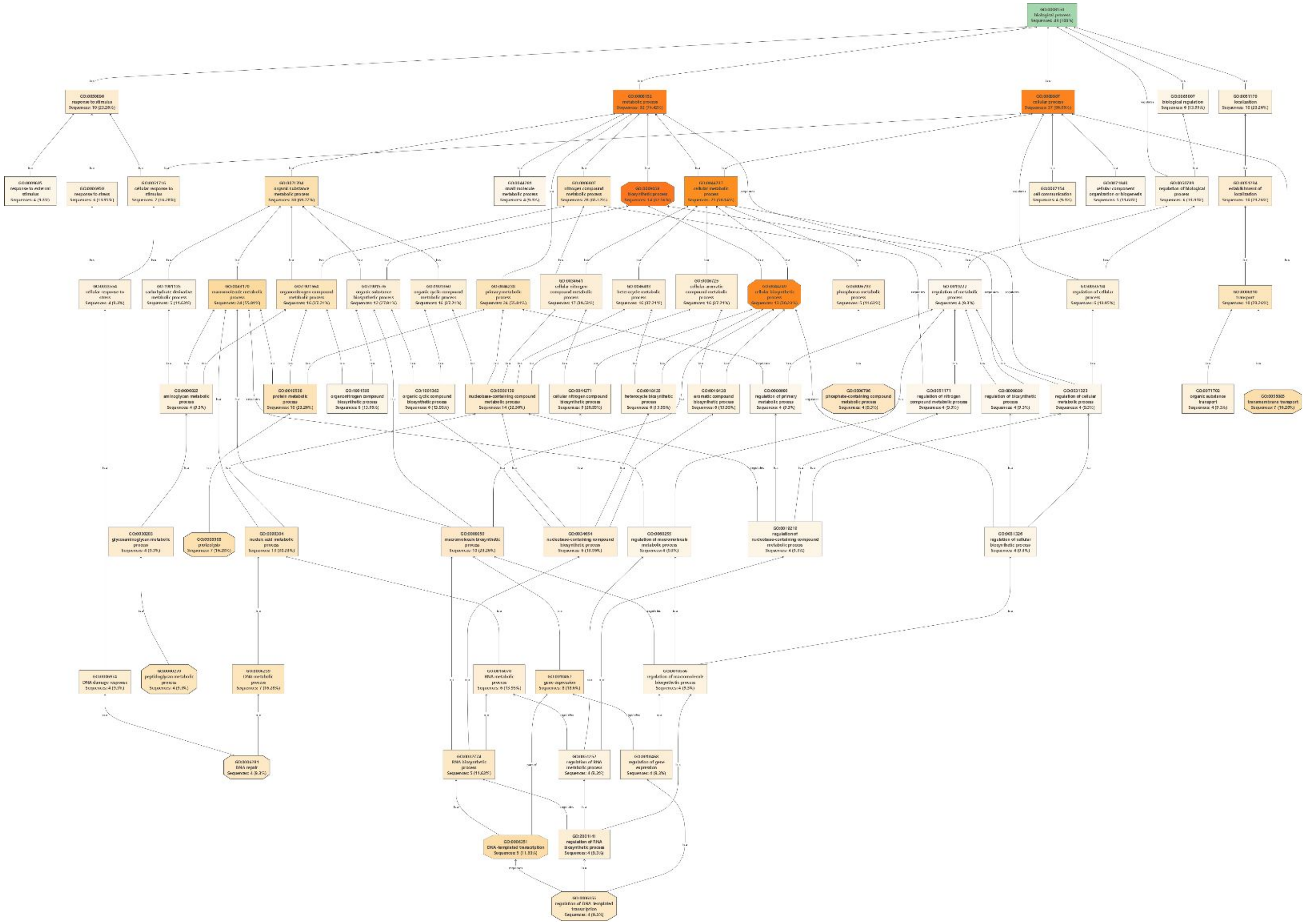

Figure S4. Combined hierarchical graph displaying *Rhizobium tropici* CIAT 899 proteins identified in the membrane vesicles isolated from the peribacteroid space of *Lotus burttii* nodules and distributed according to gene ontology category enrichment and classified into the biological process affected. The boxes with darker shades represent the Blast2Go categories with higher representation according to the percentage of proteins encountered.

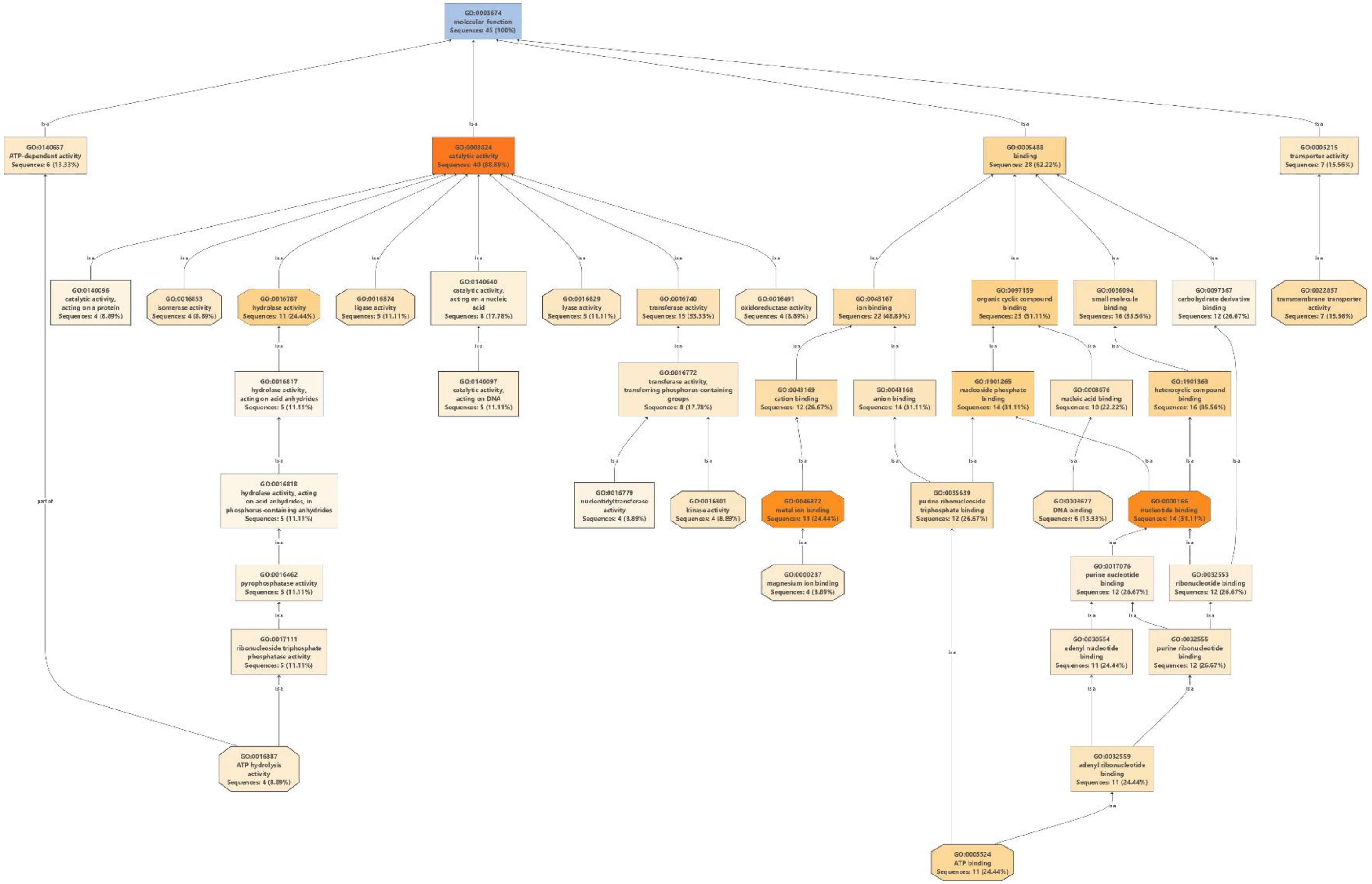

Figure S5. Combined hierarchical graph displaying *Sinorhizobium fredii* HH103 proteins identified in the membrane vesicles isolated from the peribacteroid space of soybean nodules and distributed according to gene ontology category enrichment and classified into the molecular function affected. The boxes with darker shades represent the Blast2Go categories with higher representation according to the percentage of proteins encountered.

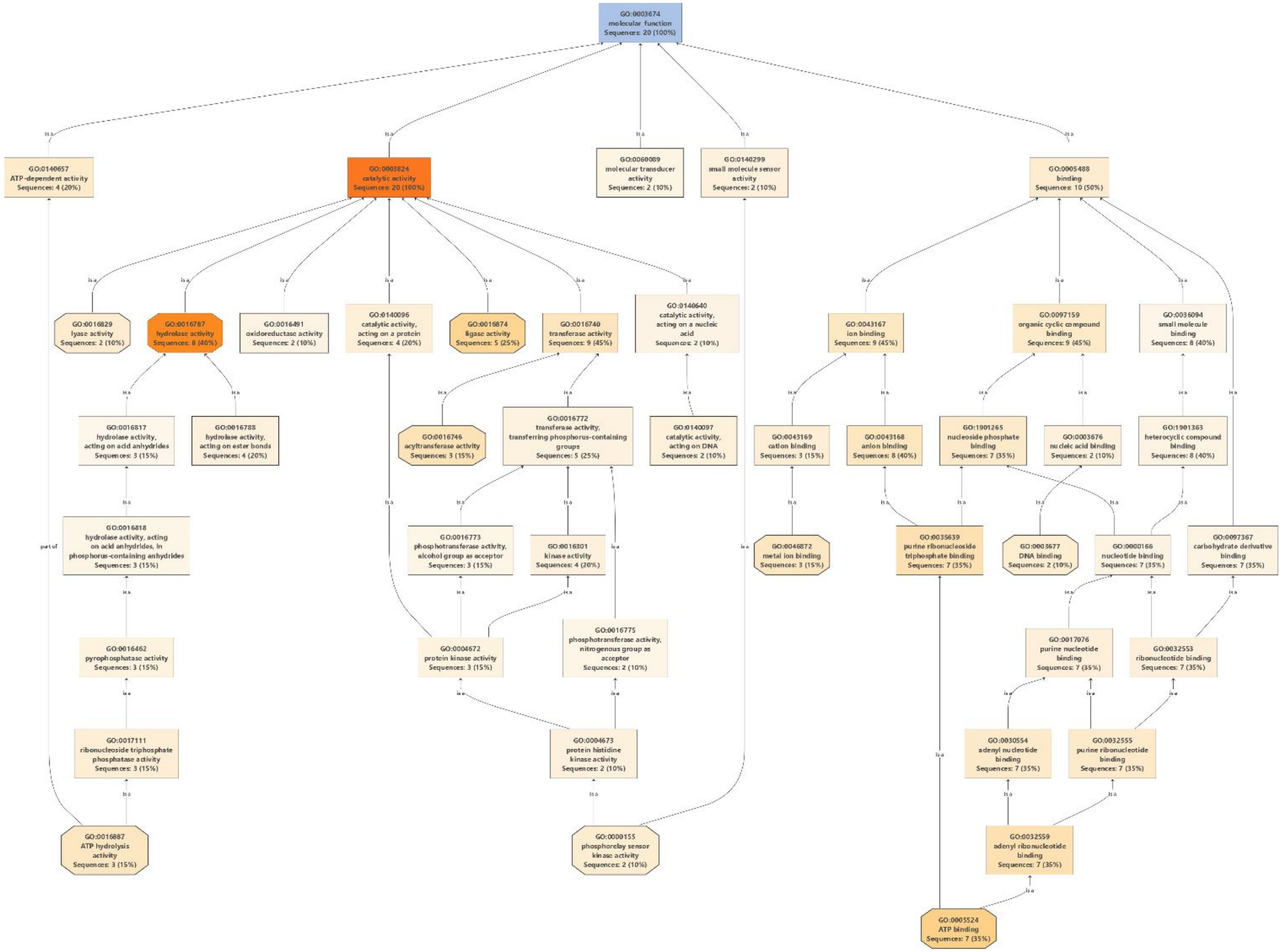

Figure S6. Combined hierarchical graph displaying *Sinorhizobium fredii* HH103 proteins identified in the membrane vesicles isolated from the peribacteroid space of *Lotus burttii* nodules and distributed according to gene ontology category enrichment and classified into the molecular function affected. The boxes with darker shades represent the Blast2Go categories with higher representation according to the percentage of proteins encountered.

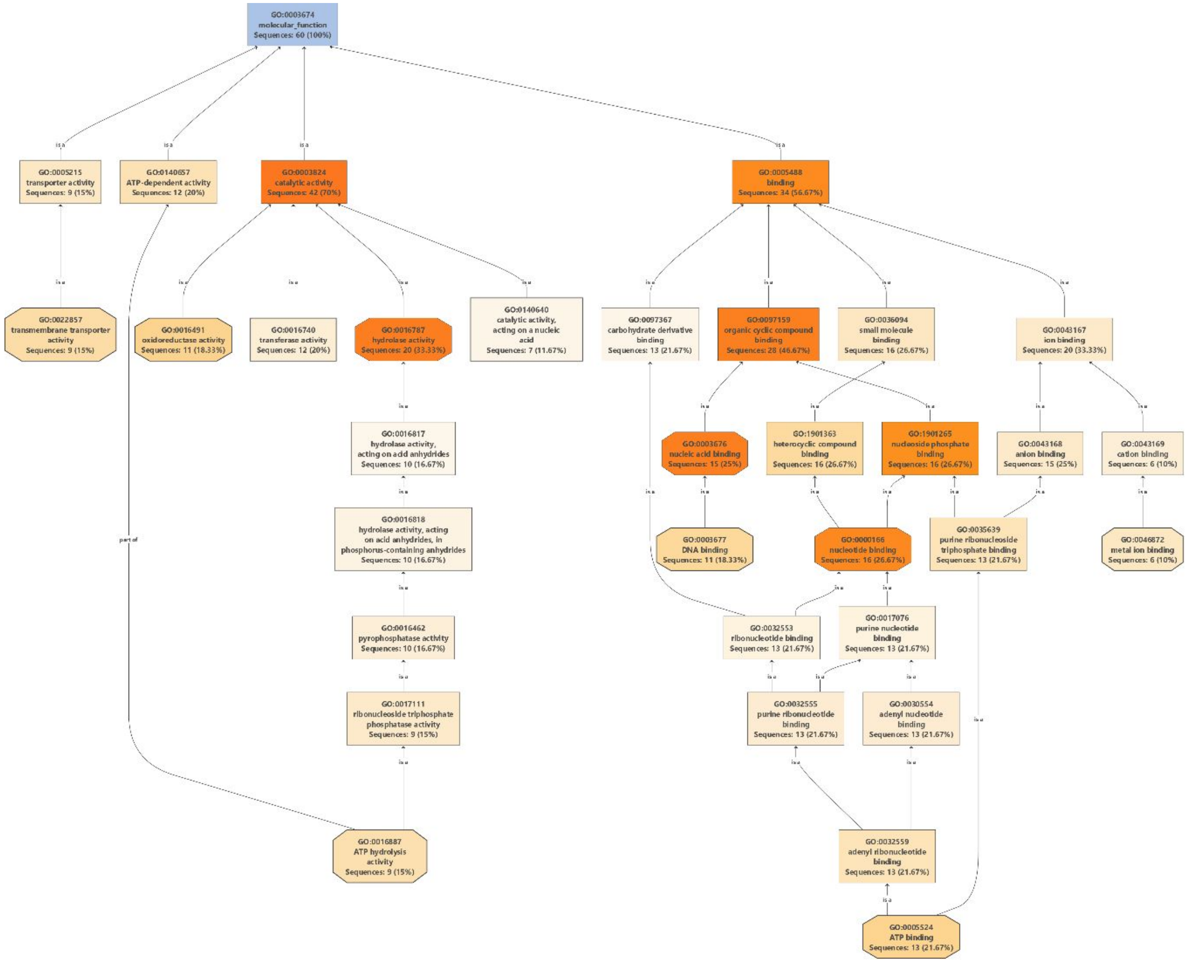

Figure S7. Combined hierarchical graph displaying *Rhizobium tropici* CIAT 899 proteins identified in the membrane vesicles isolated from the peribacteroid space of bean nodules and distributed according to gene ontology category enrichment and classified into the molecular function affected. The boxes with darker shades represent the Blast2Go categories with higher representation according to the percentage of proteins encountered.

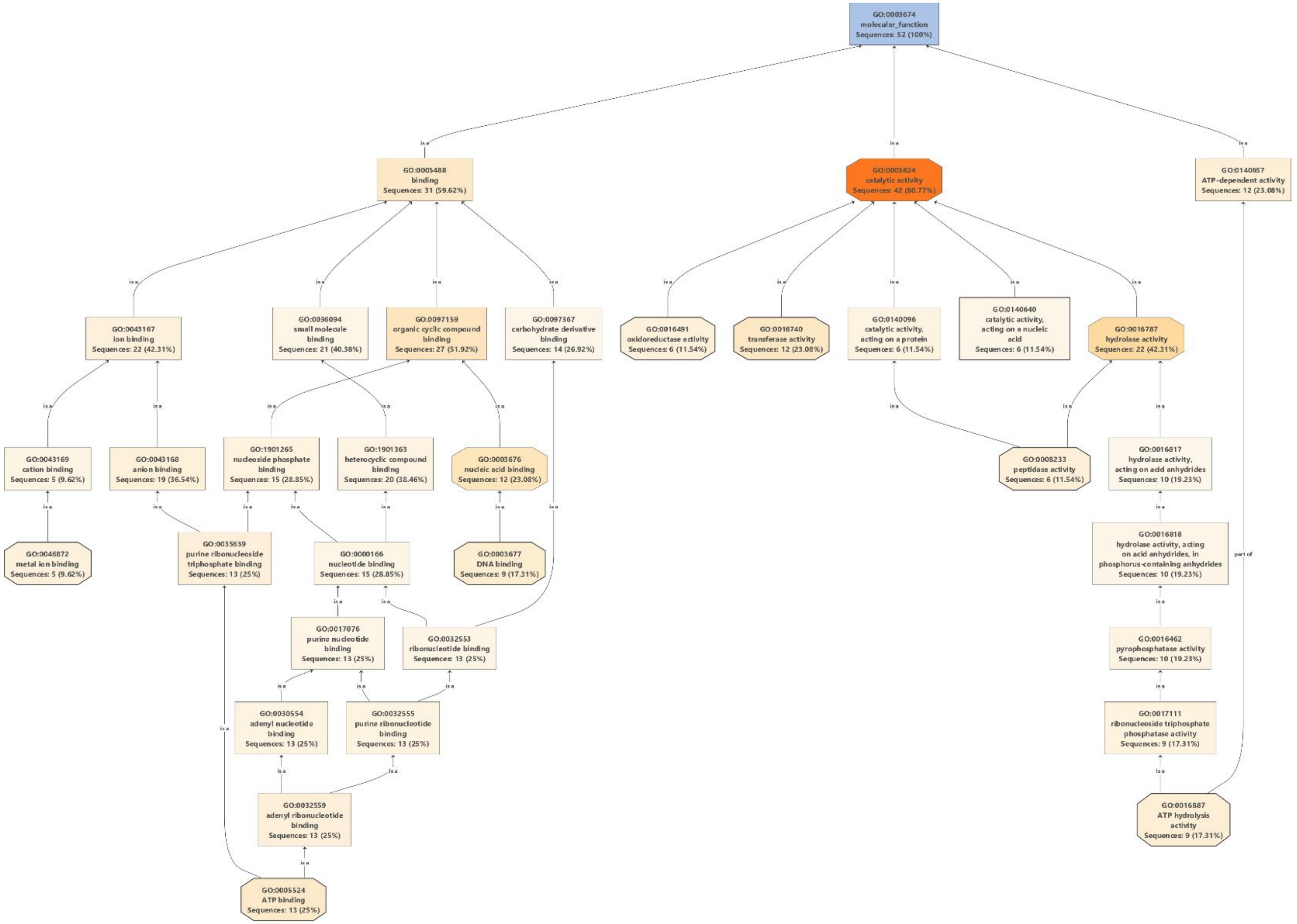

Figure S8. Combined hierarchical graph displaying *Rhizobium tropici* CIAT 899 proteins identified in the membrane vesicles isolated from the peribacteroid space of *Lotus burttii* nodules and distributed according to gene ontology category enrichment and classified into the molecular function affected. The boxes with darker shades represent the Blast2Go categories with higher representation according to the percentage of proteins encountered.

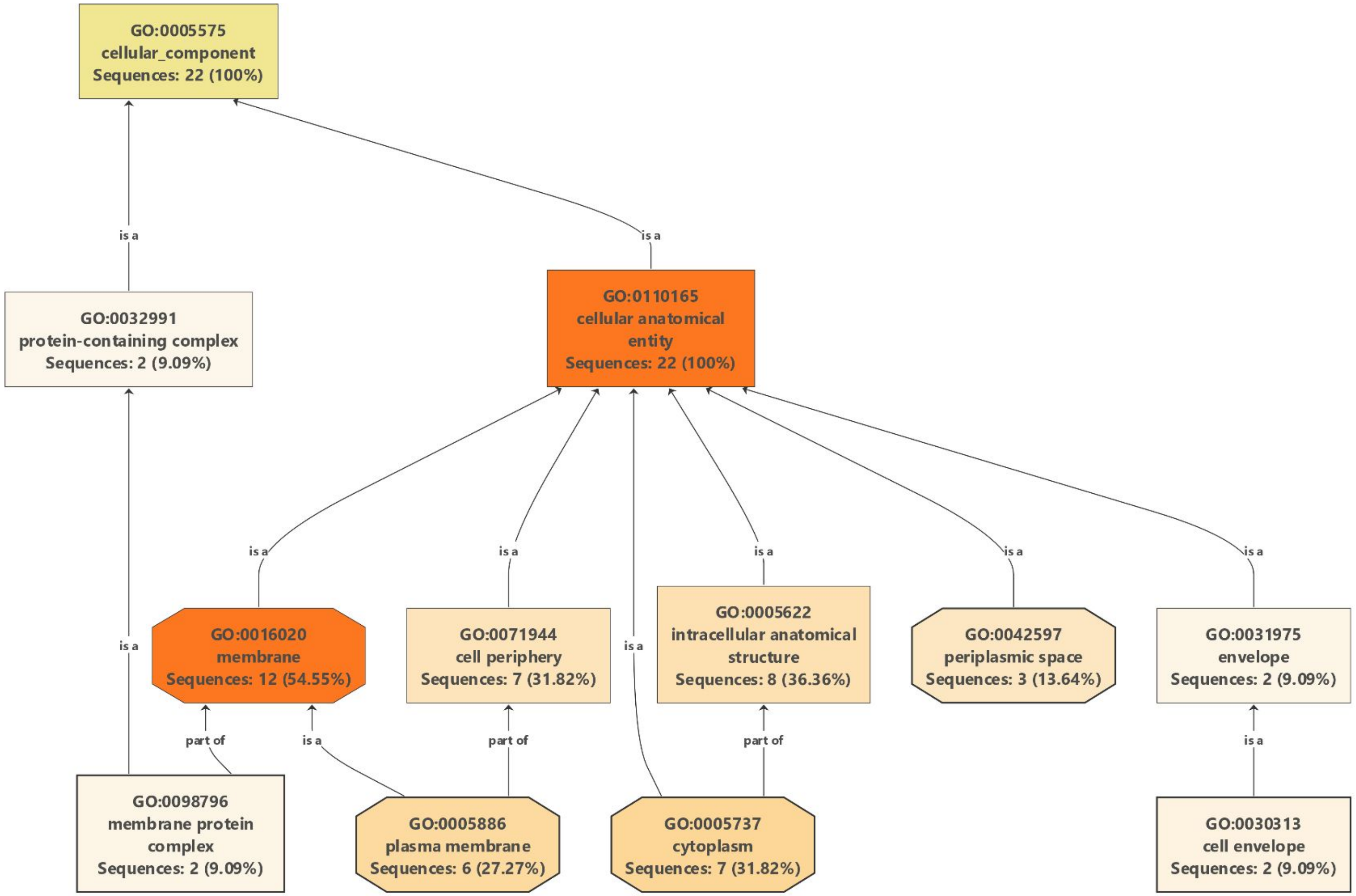

Figure S9. Combined hierarchical graph displaying *Sinorhizobium fredii* HH103 proteins identified in the membrane vesicles isolated from the peribacteroid space of soybean nodules and distributed according to gene ontology category enrichment and classified into the cellular component affected. The boxes with darker shades represent the Blast2Go categories with higher representation according to the percentage of proteins encountered.

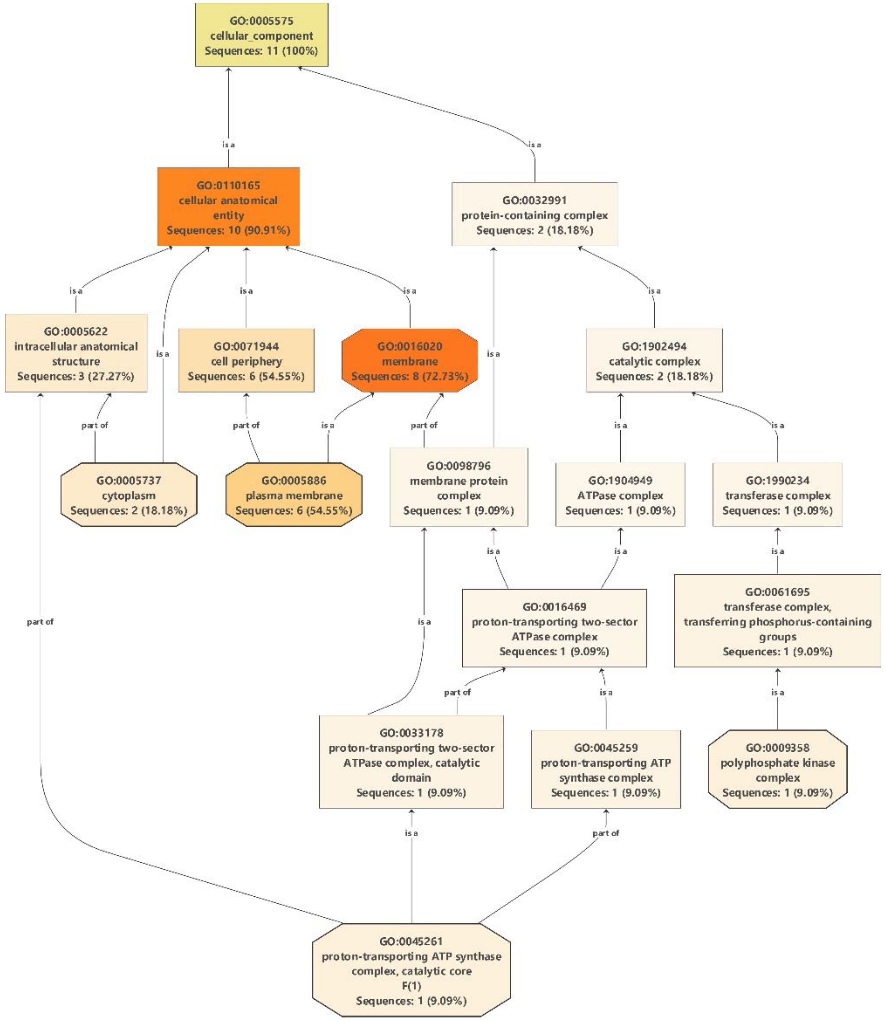

Figure S10. Combined hierarchical graph displaying *Sinorhizobium fredii* HH103 proteins identified in the membrane vesicles isolated from the peribacteroid space of *Lotus burttii* nodules and distributed according to gene ontology category enrichment and classified into the cellular component affected. The boxes with darker shades represent the Blast2Go categories with higher representation according to the percentage of proteins encountered.

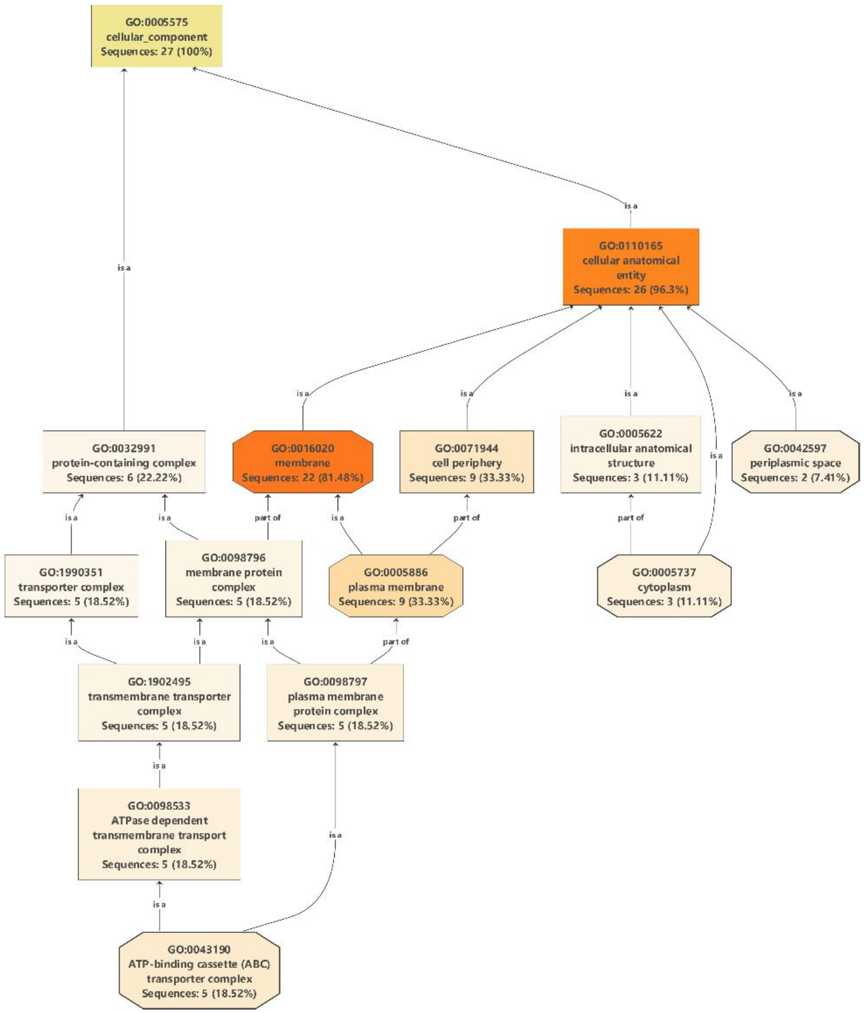

Figure S11. Combined hierarchical graph displaying *Rhizobium tropici* CIAT 899 proteins identified in the membrane vesicles isolated from the peribacteroid space of bean nodules and distributed according to gene ontology category enrichment and classified into the cellular component affected. The boxes with darker shades represent the Blast2Go categories with higher representation according to the percentage of proteins encountered.

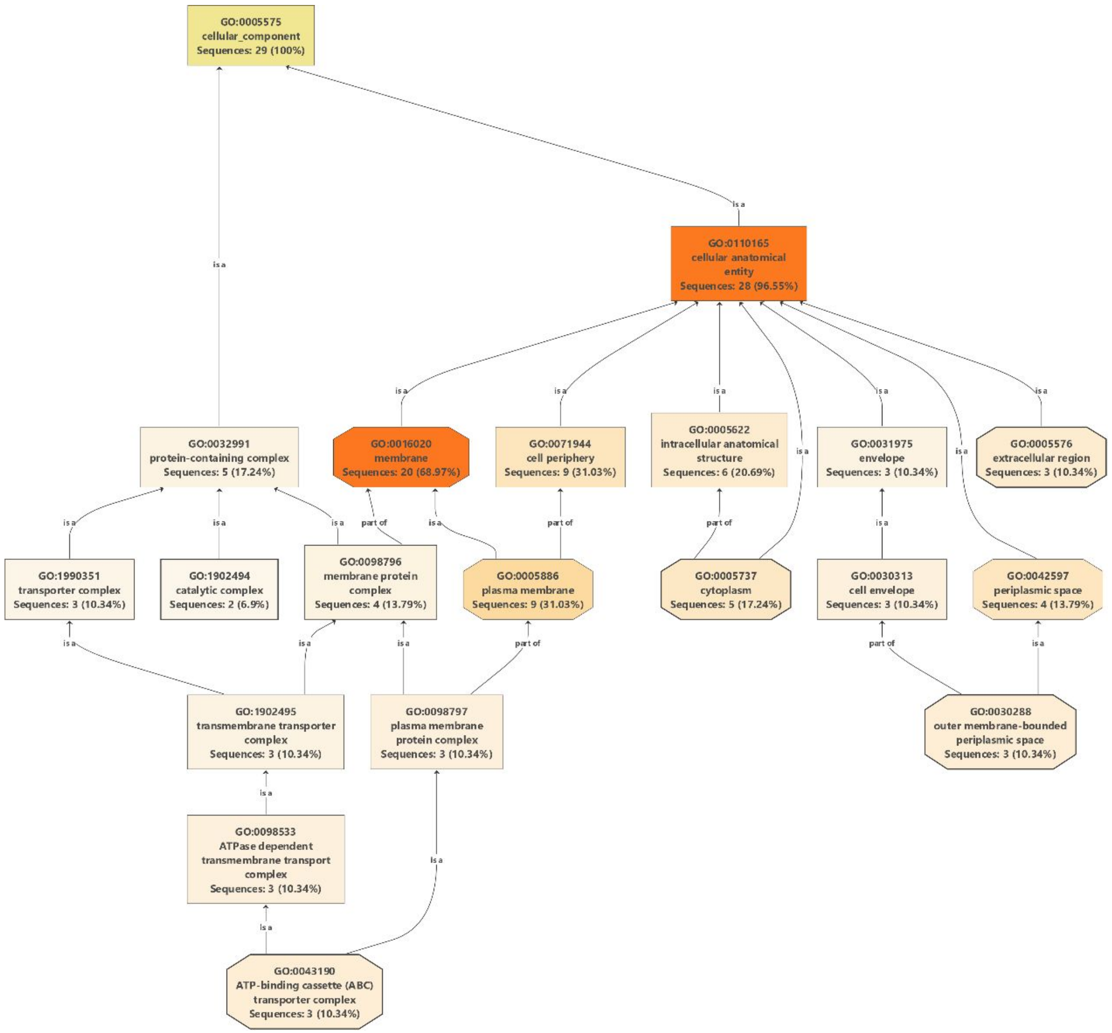

Figure S12. Combined hierarchical graph displaying *Rhizobium tropici* CIAT 899 proteins identified in the membrane vesicles isolated from the peribacteroid space of *Lotus burttii* nodules and distributed according to gene ontology category enrichment and classified into the cellular component affected. The boxes with darker shades represent the Blast2Go categories with higher representation according to the percentage of proteins encountered.
